# GWAS meta-analysis of CSF Alzheimer's disease biomarkers 18,948 individuals reveal novel loci and genes regulating lipid metabolism, brain volume and autophagy

**DOI:** 10.21203/rs.3.rs-6597595/v1

**Published:** 2025-05-21

**Authors:** Carlos Cruchaga, Jigyasha Timsina, Chenyang Jiang, Daniel McCartney, Feifei Tao, Maria Carolina Dalmasso, Jenna Najar, Federica Anastasi, Olena Ohlei, Raquel Puerta, Joseph Bradley, Daniel Western, Muhammad Ali, Ciyang Wang, Chengran Yang, Ying Wu, Menghan Liu, John Budde, Julie Williams, Rebecca Mahoney, atahualpa castillo, Timothy Hohman, Logan Dumitrescu, Ting-Chen Wang, Niccoló Tesi, Silke Kern, Margda Waern, Ingmer Skoog, Argonde van Harten, Wiesje van der Flier, Pascual Sánchez-Juan, Eloy Rodriguez-Rodriguez, Luca Kleineidam, Oliver Peters, Anja Schneider, Fahri Küçükali, Céline Bellenguez, Benjamin Grenier-Boley, Sami Heikkinen, Itziar de Rojas, Dan Rujescu, Norbert Scherbaum, Lucrezia Hausner, Emrah Duzel, Timo Grimmer, Jens Wiltfang, Rik Vandenberghe, Sebastiaan Engelborghs, Stefanie Heilmann-Heimbach, Matthias Schmid, Thomas Tegos, Nikolaos Scarmeas, Oriol Dols-Icardo, Fermin Moreno, Jordi Pérez-Tur, María J. Bullido, Raquel Sanchez-Valle, Victoria Álvarez, Pablo Garcia-Gonzalez, Pablo Mir, Luis Real, Gerard Piñol-Ripoll, Jose María García-Alberca, Harro Seelaar, Inez Ramakers, Janne Papma, Marc Hulsman, Christoph Laske, Stefan Teipel, Josef Priller, Robert Perneczky, Katharina Buerger, Markus Nöthen, Piotr Lewczuk, Johannes Kornhuber, Wolfgang Maier, Harald Hampel, Ina Giegling, Oliver Goldhardt, Janine Diehl-Schmid, Victor Andrade, Michael Heneka, Lutz Froelich, Jonathan Vogelgsang, Caroline Graff, Håkan Thonberg, Abbe Ullgren, Goran Papenberg, Anne Boland, Jean-François Deleuze, Michael Wagner, Frank Jessen, Henne Holstege, Cornelia van Duijn, Thibaud Lebouvier, Olivier Hanon, Ville Leinonen, Hilkka Soininen, Sanna-Kaisa Herukka, Vilmantas Giedraitis, Malin Löwenmark, Lena Kilander, Patricia Genius, Blanca Rodríguez, Emma Luckett, Arcadi Navarro, Amanda Cano, Marta Marquié, Kaj Blennow, Henrik Zetterberg, Alberto Lleo, Mercè Boada, Agustin Ruiz Laza, Virginia Lee, Vivianna Van Deerlin, Yuetiva Deming, Sterling Johnson, Corinne Engelman, Pau Pastor, Ignacio Alvarez, Elaine Peskind, Amanda Heslegrave, Andrew Saykin, Kwangsik Nho, Suzanne Schindler, John Morris, David Holtzman, Eric McDade, Alan Renton, Alison Goate, Laura Ibanez, Matthias Riemenschneider, Marilyn Albert, Simon Laws, Tenielle Porter, Eleanor O'Brien, Leslie Shaw, Betty Tijms, Martin Ingelsson, Pieter Jelle Visser, Mikko Hiltunen, Kristel Sleegers, Craig Ritchie, Rebecca Sims, Jean-Charles Lambert, Natàlia Vilor-Tejedor, Maria Fernández, Qingqin Li, Michael Nagle, Riccardo Marioni, Alfredo Ramirez, Lars Bertram, Sven van der Lee

## Abstract

Cerebrospinal fluid (CSF) amyloid beta (Aβ42), total tau (t-tau), and phosphorylated tau (p-tau181) are well accepted markers of Alzheimer’s disease. We performed a GWAS meta-analysis including 18,948 individuals of European and 416 non-European ancestry. We identified 12 genome-wide significant loci across all three biomarkers, eight of them novel. We replicated the association of CSF biomarkers with *APOE* , *CR1* , *GMNC/CCDC50* and *C16orf95/MAP1LC3B* . Novel loci included *BIN1* for Aβ42 and *GNA12, MS4A6A, SLCO1A2* with both t-tau and p-tau181, as well as additional loci on chr. 8, near *ANGPT1* and chr. 9 near *SMARCA2* . We also demonstrated that these variants were not only associated with CSF level of the three biomarkers but also showed significant association with AD risk, disease progression and/or brain amyloidosis. The associated genes are implicated in lipid metabolism independent *APOE* , as well as autophagy and brain volume regulation driven by t-tau and p-tau181 dysregulation.

**Carlos Cruchaga**
ccruchaga@wustl.edu

Washington University, School of Medicine https://orcid.org/0000-0002-0276-2899

**Jigyasha Timsina**
Department of Psychiatry, Washington University School of Medicine, St. Louis, MO, USA

**Chenyang Jiang**
Alzheimer Center Amsterdam, Neurology, Vrije Universiteit Amsterdam, Amsterdam UMC location VUmc

**Daniel McCartney**
University of Edinburgh

**Feifei Tao**
Human Genetics and Causal Biology, Eisai Center for Genetics-Guided Dementia Discovery, Eisai Inc, Cambridge, MA, USA

**Maria Carolina Dalmasso**
Division of Neurogenetics and Molecular Psychiatry, Department of Psychiatry and Psychotherapy, Faculty of Medicine and University Hospital Cologne, University of Cologne, Cologne, Germany

**Jenna Najar**
Alzheimer Center Amsterdam, Neurology, Vrije Universiteit Amsterdam, Amsterdam UMC location VUmc

**Federica Anastasi**
Barcelonaβeta Brain Research Center (BBRC), Pasqual Maragall Foundation, Barcelona, Spain

**Olena Ohlei**
University of Lübeck

**Raquel Puerta**
Ace Alzheimer Center Barcelona – Universitat Internacional de Catalunya. Universitat de Barcelona (UB) https://orcid.org/0000-0002-1191-5893

**Joseph Bradley**
Washington University in St. Louis https://orcid.org/0000-0003-0217-4808

**Daniel Western**
Department of Psychiatry, Washington University School of Medicine, St. Louis, MO, USA https://orcid.org/0000-0003-3725-9553

**Muhammad Ali**
Washington University School of Medicine https://orcid.org/0000-0002-1399-6631

**Ciyang Wang**
Washington University School of Medicine https://orcid.org/0000-0001-5253-0490

**Chengran Yang**
Washington University in St. Louis https://orcid.org/0000-0003-4577-5590

**Ying Wu**
Human Genetics and Causal Biology, Eisai Center for Genetics-Guided Dementia Discovery, Eisai Inc, Cambridge, MA, US

**Menghan Liu**
Washington University in St. Louis https://orcid.org/0009-0008-9902-5983

**John Budde**
https://orcid.org/0000-0001-7778-7276

**Julie Williams**
Cardiff University https://orcid.org/0000-0002-4069-0259

**Rebecca Mahoney**
UK Dementia Research Institute at Cardiff, Cardiff, UK

**atahualpa castillo**
School of Medicine, Cardiff University

**Timothy Hohman**
Vanderbilt University Medical Center https://orcid.org/0000-0002-3377-7014

**Logan Dumitrescu**
Vanderbilt University Medical Center

Vanderbilt Memory and Alzheimer's Center, Vanderbilt University Medical Center, Nashville, Tennessee, USA.
**Niccoló Tesi**
Genomics of Neurodegenerative Diseases and Aging https://orcid.org/0000-0002-1413-5091
**Silke Kern**
University of Gothenburg
**Margda Waern**
https://orcid.org/0000-0002-8330-6915
**Ingmer Skoog**
University of Gothenburg
**Argonde van Harten**
Alzheimer Center Amsterdam, Neurology, Vrije Universiteit Amsterdam, Amsterdam UMC location VUmc, Amsterdam, The Netherlands.
**Wiesje van der Flier**
Alzheimer Center Amsterdam https://orcid.org/0000-0001-8766-6224
**Pascual Sánchez-Juan**
Alzheimer's Centre Reina Sofia-CIEN Foundation-ISCIII
**Eloy Rodriguez-Rodriguez**
Marqués de Valdecilla University Hospital https://orcid.org/0000-0001-7742-677X
**Luca Kleineidam**
University of Bonn https://orcid.org/0009-0006-3309-6856
**Oliver Peters**
Department of Psychiatry and Neurosciences, Charité - Universitätsmedizin Berlin, corporate member of Freie Universität Berlin and Humboldt-Universität zu Berlin https://orcid.org/0000-0003-0568-2998
**Anja Schneider**
University of Bonn
**Fahri Küçükali**
VIB Center for Molecular Neurology https://orcid.org/0000-0002-3835-9639
**Céline Bellenguez**
Inserm https://orcid.org/0000-0002-1240-7874
**Benjamin Grenier-Boley**
Univ. Lille, Inserm, CHU Lille, Institut Pasteur de Lille, LabEx DISTALZ - U1167-RID-AGE Facteurs de risque et déterminants moléculaires des maladies liées au vieillissement, Li
**Sami Heikkinen**
Institute of Biomedicine, School of Medicine, University of Eastern Finland, Kuopio Campus https://orcid.org/0000-0002-6083-2402
**Itziar de Rojas**
Research Center and Memory clinic, ACE Alzheimer Center Barcelona, Universitat Internacional de Catalunya, Spain https://orcid.org/0000-0002-2148-381X
**Dan Rujescu**
Medical University of Vienna https://orcid.org/0000-0002-1432-313X
**Norbert Scherbaum**
University of Duisburg-Essen https://orcid.org/0000-0003-1759-6990
**Lucrezia Hausner**
University of Heidelberg https://orcid.org/0000-0001-9009-2356
**Emrah Duzel**
German Center for Neurodegenerative Diseases
**Timo Grimmer**
Center for Cognitive Disorders, Department of Psychiatry and Psychotherapy, Technical University of Munich, School of Medicine, Munich, Germany
**Jens Wiltfang**
Universitätsmedizin Göttingen https://orcid.org/0000-0003-1492-5330
**Rik Vandenberghe**
Department of Neurology, UZ Leuven, Leuven, Belgium.
**Sebastiaan Engelborghs**
Antwerp University https://orcid.org/0000-0003-0304-9785
**Stefanie Heilmann-Heimbach**
School of Medicine & University Hospital Bonn https://orcid.org/0000-0003-1057-465X
**Matthias Schmid**
German Center for Neurodegenerative Diseases (DZNE)
**Thomas Tegos**
Aristotle University of Thessaloniki
**Nikolaos Scarmeas**

Taub Institute for Research in Alzheimer’s Disease and the Aging Brain
**Oriol Dols-Icardo**
Institut de recerca de l'hospital de Sant Pau https://orcid.org/0000-0003-2656-8748
**Fermin Moreno**
Hospital Universitario Donostia https://orcid.org/0000-0001-5200-3164
**Jordi Pérez-Tur**
Institut de Biomedicina de València-CSIC https://orcid.org/0000-0002-9111-1712
**María J. Bullido**
Centro de Biología Molecular Severo Ochoa https://orcid.org/0000-0002-6477-1117
**Raquel Sanchez-Valle**
Hospital Clinic of Barcelona
**Victoria Álvarez**
Hospital Universitario Central de Asturias https://orcid.org/0000-0002-1916-2523
**Pablo Garcia-Gonzalez**
Ace Alzheimer Center Barcelona https://orcid.org/0000-0003-0125-5403
**Pablo Mir**
Hospital Universitario Virgen del Rocío/CSIC/Universidad de Sevilla https://orcid.org/0000-0003-1656-302X
**Luis Real**
Unidad Clínica de Enfermedades Infecciosas y Microbiología. Hospital Universitario de Valme
**Gerard Piñol-Ripoll**
Santa Maria University Hospital-IRBLleida
**Jose María García-Alberca**
Andalusian Institute for Neuroscience https://orcid.org/0000-0003-2951-6644
**Harro Seelaar**
https://orcid.org/0000-0003-1989-7527
**Inez Ramakers**
Maastricht University
**Janne Papma**
Erasmus MC University Medical Center
**Marc Hulsman**
Amsterdam UMC https://orcid.org/0000-0002-9889-3606
**Christoph Laske**
German Center for Neurodegenerative Diseases (DZNE), Tübingen, Germany
**Stefan Teipel**
German Center for Neurodegenerative Diseases (DZNE), Rostock, Germany
**Josef Priller**
University of Berlin
**Robert Perneczky**
University Hospital of Munich, LMU Munich https://orcid.org/0000-0003-1981-7435
**Katharina Buerger**
Ludwig Maximilian University of Munich
**Markus Nöthen**
School of Medicine & University Hospital Bonn https://orcid.org/0000-0002-8770-2464
**Piotr Lewczuk**
Universitätsklinikum Erlangen, and Friedrich-Alexander Universität Erlangen-Nürnberg
**Johannes Kornhuber**
Friedrich-Alexander University of Erlangen https://orcid.org/0000-0002-8096-3987
**Wolfgang Maier**
University of Bonn
**Harald Hampel**
Sorbonne University, Alzheimer Precision Medicine (APM), AP-HP, Pitié-Salpêtrière Hospital, Boulevard de l'hôpital, Paris, France
**Ina Giegling**
Division of General Psychiatry, Dept. of Psychiatry and Psychotherapy, Medical University of Vienna, Vienna, Austria
**Oliver Goldhardt**
Technische Universitat Munchen
**Janine Diehl-Schmid**
**Victor Andrade**
University of cologne
**Michael Heneka**

Department of Neurodegenerative Diseases and Geriatric Psychiatry, University Hospital Bonn, Medical Faculty, Bonn, Germany
**Lutz Froelich**
Central Institute of Mental Health, University of Heidelberg https://orcid.org/0000-0003-1494-0813
**Jonathan Vogelgsang**
Universitatsklinikum Gottingen https://orcid.org/0000-0001-9326-8193
**Caroline Graff**
Karolinska Institutet
**Håkan Thonberg**
Karolinska Institutet, Karolinska University Hospital
**Abbe Ullgren**
Karolinska Institutet
**Goran Papenberg**
Aging Research Center
**Anne Boland**
Université Paris-Saclay, CEA, Centre National de Recherche en Génomique Humaine https://orcid.org/0000-0001-8789-5676
**Jean-François Deleuze**
Centre National de Recherche en Génomique Humaine
**Michael Wagner**
German Center for Neurodegenerative Diseases https://orcid.org/0000-0003-2589-6440
**Frank Jessen**
**Henne Holstege**
Amsterdam UMC Location VUmc https://orcid.org/0000-0002-7688-3087
**Cornelia van Duijn**
University of Oxford https://orcid.org/0000-0002-2374-9204
**Thibaud Lebouvier**
Univ Lille Inserm 1171, CHU Clinical and Research Memory Research Centre (CMRR) of Distalz Lille France.
**Olivier Hanon**
Université de Paris, EA 4468, APHP, Hôpital Broca
**Ville Leinonen**
University of Eastern Finland and Department of Neurosurgery, Kuopio University Hospital https://orcid.org/0000-0002-4282-4687
**Hilkka Soininen**
University of Eastern Finland https://orcid.org/0000-0002-2785-9937
**Sanna-Kaisa Herukka**
Department of Neurology, Institute of Clinical Medicine, University of Eastern Finland, Kuopio, Finland.
**Vilmantas Giedraitis**
Department of Public Health and Caring Sciences, Clinical Geriatrics, Uppsala University
**Malin Löwenmark**
Department of Public Health and Caring Sciences/Clinical Geriatrics, Uppsala University, Uppsala, Sweden
**Lena Kilander**
Dept. of Public Health and Caring Sciences/Geriatrics
**Patricia Genius**
Barcelonaβeta Brain Research Center (BBRC), Pasqual Maragall Foundation, Barcelona, Spain
**Blanca Rodríguez**
Barcelonaβeta Brain Research Center (BBRC), Pasqual Maragall Foundation, Barcelona, Spain
**Emma Luckett**
Amsterdam UMC location VUmc, department of Radiology and Nuclear Medicine, De Boelelaan 1117, 1081 HV, Amsterdam, Netherlands
**Arcadi Navarro**
Barcelonaβeta Brain Research Center (BBRC), Pasqual Maragall Foundation, Barcelona, Spain
**Amanda Cano**
Fundacio ACE
**Marta Marquié**
Universitat Internacional de Catalunya
**Kaj Blennow**
University of Gothenburg https://orcid.org/0000-0002-1890-4193
**Henrik Zetterberg**
University of Gothenburg https://orcid.org/0000-0003-3930-4354
**Alberto Lleo**
Universitat Autònoma de Barcelona https://orcid.org/0000-0002-2568-5478
**Mercè Boada**

Universitat Internacional de Catalunya https://orcid.org/0000-0003-2617-3009
**Agustin Ruiz Laza**
ACE Alzheimer Center Barcelona https://orcid.org/0000-0003-2633-2495
**Virginia Lee**
University of Pennsylvania https://orcid.org/0000-0003-3536-6902
**Vivianna Van Deerlin**
Department of Pathology and Laboratory Medicine, Perelman School of Medicine at the University of Pennsylvania, Philadelphia, PA, USA 19104
**Yuetiva Deming**
Wisconsin Alzheimer's Disease Research Center, University of Wisconsin School of Medicine and Public Health, University of Wisconsin-Madison, Madison, WI, USA
**Sterling Johnson**
wisc https://orcid.org/0000-0002-8501-545X
**Corinne Engelman**
University of Wisconsin-Madison
**Pau Pastor**
Fundació Docència i Recerca MútuaTerrassa https://orcid.org/0000-0002-7493-8777
**Ignacio Alvarez**
Germans Trias i Pujol Research Institute (IGTP) https://orcid.org/0000-0002-8537-3935
**Elaine Peskind**
Department of Veterans Affairs, Mental Illness Research Education and Clinical Center
**Amanda Heslegrave**
UK Dementia Research Institute at UCL, London, UK
**Andrew Saykin**
Indiana University School of Medicine https://orcid.org/0000-0002-1376-8532
**Kwangsik Nho**
Indiana Alzheimer's Disease Research Center, Department of Radiology and Imaging Sciences, Indiana University School of Medicine, Indianapolis, Indiana USA
**Suzanne Schindler**
Washington University School of Medicine https://orcid.org/0000-0002-1680-1465
**John Morris**
Knight Alzheimer Disease Research Center
**David Holtzman**
Washington University in St Louis School of Medicine https://orcid.org/0000-0002-3400-0856
**Eric McDade**
Department of Neurology, Washington University School of Medicine, St. Louis, MO, USA
**Alan Renton**
Nash Family Department of Neuroscience and Ronald Loeb Center for Alzheimer's Disease, Icahn School of Medicine at Mount Sinai, New York, NY, USA: Departments of Neurology and Genetics and Ge https://orcid.org/0000-0001-6702-8268
**Alison Goate**
Mount Sinai School of Medicine https://orcid.org/0000-0002-0576-2472
**Laura Ibanez**
Washington University in St. Louis
**Matthias Riemenschneider**
Klinik für Psychiatrie und Psychotherapie
**Marilyn Albert**
Johns Hopkins School of Medicine
**Simon Laws**
Edith Cowan University https://orcid.org/0000-0002-4355-7082
**Tenielle Porter**
Edith Cowan University https://orcid.org/0000-0002-7887-6622
**Eleanor O'Brien**
Edith Cowan University https://orcid.org/0000-0001-5145-7340
**Leslie Shaw**
Department of Pathology and Laboratory Medicine, Perelman School of Medicine at the University of Pennsylvania, Philadelphia, PA, USA
**Betty Tijms**
Amsterdam UMC, location VUmc https://orcid.org/0000-0002-2612-1797
**Martin Ingelsson**
Uppsala University https://orcid.org/0000-0001-5466-8370
**Pieter Jelle Visser**

AmsterdamUMC location VUmc https://orcid.org/0000-0001-8008-9727
**Mikko Hiltunen**
Institute of Biomedicine, University of Eastern Finland, Kuopio, Finland
**Kristel Sleegers**
Department of Biomedical Sciences, University of Antwerp, Antwerp, Belgium
**Craig Ritchie**
Scottish Brain Sciences, Scottish Gas Murrayfield Stadium, Edinburgh, UK
**Rebecca Sims**
Cardiff University https://orcid.org/0000-0002-3885-1199
**Jean-Charles Lambert**
Pasteur Institute of Lille https://orcid.org/0000-0003-0829-7817
**Natàlia Vilor-Tejedor**
Hospital del Mar Medical Research Institute
**Maria Fernández**
Washington University School of Medicine
**Qingqin Li**
Neuroscience, Janssen Research & Development, LLC, Titusville, NJ, 08560, USA
**Michael Nagle**
Eisai https://orcid.org/0000-0002-4677-7582
**Riccardo Marioni**
University of Edinburgh https://orcid.org/0000-0003-4430-4260
**Alfredo Ramirez**
University Hospital Cologne https://orcid.org/0000-0003-4991-763X
**Lars Bertram**
University of Lübeck https://orcid.org/0000-0002-0108-124X
**Sven van der Lee**
Amsterdam UMC, location VUmc https://orcid.org/0000-0003-1606-8643

**Article**

**Keywords:**

**Posted Date:** May 21st, 2025

**DOI:** https://doi.org/10.21203/rs.3.rs-6597595/v1

**License:** This work is licensed under a Creative Commons Attribution 4.0 International License. Read Full License

**Additional Declarations: Yes** there is potential Competing Interest. COI, where applicable, are listed in manuscript.

## Abstract

Cerebrospinal fluid (CSF) amyloid beta (Aβ42), total tau (t-tau), and phosphorylated tau (p-tau181) are well accepted markers of Alzheimer's disease. We performed a GWAS meta-analysis including 18,948 individuals of European and 416 non-European ancestry. We identified 12 genome-wide significant loci across all three biomarkers, eight of them novel. We replicated the association of CSF biomarkers with *APOE*, *CR1*, *GMNC/CCDC50* and *C16orf95/MAP1LC3B*. Novel loci included *BIN1* for Aβ42 and *GNA12, MS4A6A, SLCO1A2* with both t-tau and p-tau181, as well as additional loci on chr. 8, near *ANGPT1* and chr. 9 near *SMARCA2*. We also demonstrated that these variants were not only associated with CSF level of the three biomarkers but also showed significant association with AD risk, disease progression and/or brain amyloidosis. The associated genes are implicated in lipid metabolism independent *APOE*, as well as autophagy and brain volume regulation driven by t-tau and p-tau181 dysregulation.

## Introduction

Alzheimer's disease (AD), a progressive neurodegenerative disease, is the most common cause of dementia worldwide[1]. Decades of research have identified mutations in *APP, PSEN1*, and *PSEN2* as the genetic basis for the familial form of AD[2,3]. For the more common sporadic AD and related dementias, genetic studies have identified more than 74 loci associated with the disease [4–8]. However, most of the genetic studies in AD are focused on clinical and symptomatic status, which can be biased due to the presence of presymptomatic subjects. Incorrect or mixed dementia diagnosis further add to these challenges. Endophenotypes are measurable traits that serve as intermediates between genetic variants and disease, providing a closer link to the disease processes involved. Since endophenotypes can often be more directly linked to genetic variation, their associations may be easier to interpret and better reflect the underlying mechanisms of disease.

Cerebrospinal fluid (CSF) Aβ42, total tau (t-tau) and phosphorylated tau (p-tau181) are critical and well validated core AD biomarkers and endophenotypic measures[9]. A decrease in CSF Aβ42 and an increase in CSF t-tau and p-tau181 reflects the accumulation of aggregated β-amyloid into plaques the brain, with the later also capturing neurofibrillary tangle formation to some extent and can be detected before clinical symptoms. Both processes are hallmarks of AD pathology and contribute to disease mechanisms as proposed by the amyloid cascade theory[9,10]. Thus, identifying variants that modulate the levels of these biomarkers may help to prioritize genes linked with AD pathogenesis.

Previous GWAS focusing on these three CSF endophenotypes have highlighted important genetic associations[5,11,12]. Deming *et al.*[11] identified five genome-wide significant loci associated with p-tau181 and three with Aβ42 from a GWAS of 3,146 participants across nine studies. They also demonstrated the role of these variants in disease risk and progression. Similarly, Jansen *et al.*[5] identified two loci associated with Aβ42 and four loci associated with p-tau181. Only the *APOE* and *GMNC* loci (for p-tau181) were consistently identified in both studies. Therefore, further analysis with a larger subject size has the potential to identify novel associations and provide replication of previously reported findings.

Here, we performed a GWAS encompassing CSF measures of Aβ42, t-tau and p-tau181in 18,948 individuals, across 30 different studies. These include samples used in previous GWAS by Deming *et al.*[11] and Jansen *et al*[5]. We determined 12 unique significantly associated genetic variants across the three biomarkers and performed fine mapping and functional gene nomination of the loci. In addition, we investigated the association of lead variants with AD progression, AD risk and other AD-related phenotypes. Finally, through pathway analysis, using genes linked to our variants, we investigated biological processes associated with these variants influencing our CSF biomarkers of interest.

## Results

### Meta-analysis replicates previously reported associations for CSF Aβ42, t-tau, and p-tau181

We associated individual-level genetic data with CSF biomarker levels in 18,948 unrelated European ancestry individuals from 30 different studies (Table 1). Detailed study workflow is presented in Fig. 1. We identified 12 unique genome-wide significant variants across Aβ42, t-tau, and p-tau181 including one novel association for Aβ42, five for t-tau, and four for p-tau181 (Fig. 2A; Table 2, Supplementary Fig. 1–7).

Table 1
Demographic information of subjects with CSF biomarkers used in Meta-Analysis

| Cohort | Platform | N | Average Age (SD) | %Male | Average Aβ42 | Average t-tau | Average ptau181 | %Cases | %Controls |
|---|---|---|---|---|---|---|---|---|---|
| ADNI | xMAP | 1145 | 73.59 (± 7.24) | 55.9 | 172.84 | 90.8 | 39.54 | 71.27 | 28.65 |
| AIBL | Innotest | 209 | 73.2 (± 6.14) | 50.72 | 810.77 | 285.73 | 57.86 | 27.75 | 72.25 |
| BIOCARD | NA | 167 | 60.02 (± 7.69) | 41.32 | 417.73 | 69.33 | 36.07 | 2.40 | 94.61 |
| Blennow | NA | 56 | 83.2 (± 10.21) | 26.79 | 550.91 | 679.54 | 74.14 | 58.93 | 41.97 |
| Barcelona-1 | ELISA | 279 | 67.78 (± 8.33) | 49.46 | 927.12 | 472.28 | 70.17 | 24.73 | 1.43 |
| DIAN | Lumipulse | 188 | 37.26 (± 10.12) | 42.55 | 679.02 | 408.41 | 58.73 | 22.34 | 77.66 |
| DOD | Elecsys | 101 | 68.42 (± 3.59) | 99.01 | 1216.85 | 218.96 | 19.11 | 27.72 | 69.31 |
| HB | NA | 100 | 67.35 (± 9.25) | 55 | 76.08 | 84.95 | NA | 100 | 0 |
| Lleo | NA | 122 | 63.72 (± 9.68) | 32.79 | 642.59 | 385.72 | 56.68 | 31.97 | 54.92 |
| London | NA | 253 | 68.67 (± 8.96) | 53.36 | 387.66 | 97.32 | 41.93 | 63.36 | 8.30 |
| MAP | Lumipulse | 1024 | 69.63 (± 9.44) | 47.56 | 769.38 | 387.31 | 50.21 | 29.10 | 70.80 |
| MARS | NA | 103 | 64.69 (± 8.67) | 59.22 | 834.88 | 222.38 | 37.96 | 84.47 | 15.53 |
| MAYO | NA | 425 | 78.21 (± 6.24) | 60.24 | 330.76 | 98.65 | 21.65 | 18.82 | 79.53 |
| Moli | NA | 228 | 64.68 (± 8.01) | 38.16 | 554.34 | 471.08 | 79.05 | 67.54 | 21.93 |
| NACC | Multiple | 469 | 71.37 (± 9.00) | 46.7 | 334.1 | 291.01 | 56.78 | 42.64 | 29.85 |
| PPMIS | NA | 813 | 62.17 (± 9.67) | 61.87 | 927.67 | 176.67 | 15.52 | 47.60 | 21.89 |
| SWEDEN | NA | 289 | 75.15 (± 7.66) | 37.02 | 262.94 | 783.72 | 106.01 | 100 | 0 |
| UPENN | Luminex | 46 | 72.13 (± 8.27) | 60.87 | 141.84 | 118.21 | 40.74 | 10.87 | 2.17 |
| UW | NA | 343 | 62.41 (± 15.96) | 51.31 | 141.13 | 62.95 | 56.71 | 34.40 | 59.18 |
| VMAP | NA | 135 | 72.39 (± 6.42) | 68.89 | 704.9 | 424.59 | 61.87 | 37.04 | 52.59 |
| WiscADRC | NA | 214 | 64.11 (± 9.60) | 39.25 | 669.25 | 375.87 | 49.6 | 22.89 | 76.17 |
| Zetter | NA | 76 | 73.45 (± 4.14) | 50 | 619.64 | 431.59 | 76.29 | 39.47 | 23.68 |
| ACE | Lumipulse; ELISA | 1302 | 72.51 (± 8.98) | 42.63 | 767.68 | 515.87 | 79.6 | 28.34 | 67.66 |
| ALFA+ | NeuroToolKit | 386 | 61.14 (± 4.633) | 38.34 | 1315.43 | 195.87 | 15.87 | NA | 100 |
| EADB | Multiple | 7516 | 70.96 (± 7.65) | 46.26 | 637.21 | 561.67 | 75.01 | 60.36 | 38 |
| MISSION-AD | Lumipulse | 462 | 71.71 (± 7.22) | 53.90 | 739.20 | 500.39 | 67.87 | 100 | 0 |
| EMIF | NA | 670 | 69.29 (± 8.3) | 47.9 | 309.13 | NA | NA | 23.3 | 18.2 |
| EPAD | | 1370 | 65.63 (± 7.33) | 44.05 | 1380.84 | 225.73 | 19.76 | 0 | 100 |

| Cohort | Platform | N | Average Age (SD) | %Male | Average Aβ42 | Average t-tau | Average ptau181 | %Cases | %Controls |
|---|---|---|---|---|---|---|---|---|---|
| **Janssen Cohort 1** | NA | 189 | 69.42 (± 6.33) | 45 | 451.29 | 546.46 | 69.16 | 100 | 0 |
| **Janssen Cohort 2** | NA | 268 | 71.27 (± 8.73) | 48.30 | 661.55 | 707.17 | 104.91 | 100 | 0 |

NA = Not available; N = Number of subjects; Age = Age at CSF draw in years; SD = Standard Deviation. Cases include AD cases, MCI or PD cases (if known PD cohort) only. Average Biomarker level in pg/ml or similar unit. Details on individual cohort is available as supplementary material. EADB included 12 individual cohorts; detail of which is provided in Supplementary Table 1.

Table 2

Significant Meta-analysis variants for CSF phenotypes, with results from most recent AD risk, AD progression and Amyloid imaging stu

| | | | | | | | | Metaanalysis | | AD Risk[4] | | AD Progressio | |
|---|---|---|---|---|---|---|---|---|---|---|---|---|---|
| MarkerName | rsID | Trait | EA | MAF | Closest Gene | Nominated gene | Status | Effect | P | OR | P | Effect | [illegible] |
| chr1:207625371:T:G | rs10779336 | Aβ42 | T | 0.198 | *CR1* | *CR1* | Novel | -0.073 | **3.49E-08** | 1.127 | **4.69E-31** | 4.08E-05 | [illegible] |
| **chr2:127135234:C:T** | rs6733839 | Aβ42 | T | 0.394 | *BIN1* | *BIN1* | Reported | -0.06 | **8.29E-09** | 1.184 | **6.48E-90** | 1.18E-04 | [illegible] |
| chr19:44908684:T:C | rs429358 | Aβ42 | C | 0.228 | *APOE* | -- | Reported | -0.589 | **1.90e-674** | NA | NA | 4.77E-05 | [illegible] |
| chr2:127135234:C:T | rs6733839 | t-tau | T | 0.394 | *BIN1* | *BIN1* | Reported | 0.06 | **5.24E-09** | 1.184 | **6.48E-90** | 1.18E-04 | [illegible] |
| chr3:190953006:C:T | rs35327527 | t-tau | T | 0.369 | *GMNC* | *CCDC50* | Reported | 0.151 | **3.45E-48** | 1.009 | 3.00E-01 | 2.07E-05 | [illegible] |
| **chr7:2761908:G:A** | rs798490 | t-tau | A | 0.298 | *AMZ1-GNA12* | *GNA12* | Novel | -0.074 | **1.87E-11** | 1.02 | **2.51E-02** | 1.93E-05 | [illegible] |
| **chr8:107381731:A:G** | rs1654723 | t-tau | A | 0.411 | *ANGPT1* | -- | Novel | 0.056 | **4.41E-08** | 1.005 | 5.85E-01 | 5.89E-05 | [illegible] |
| **chr9:2197987:A:G** | rs57263785 | t-tau | G | 0.248 | *SMARCA2* | -- | Novel | 0.087 | **1.12E-13** | 1.014 | 1.54E-01 | 6.20E-06 | [illegible] |
| **chr11:60207692:G:A** | rs7928895 | t-tau | A | 0.393 | *MS4A4E* | *MS4A6A* | Novel | -0.06 | **5.35E-09** | 0.928 | **2.08E-19** | -8.97E-05 | [illegible] |
| **chr12:21279895:C:T** | rs11045930 | t-tau | T | 0.288 | *SLCO1A2* | *SLCO1A2* | Novel | 0.065 | **3.26E-09** | 1.01 | 2.86E-01 | 4.37E-05 | [illegible] |
| chr16:87199910:G:A | rs4843552 | t-tau | G | 0.409 | *C16orf95* | *MAP1LC3B* | Reported | 0.085 | **1.30E-16** | 0.982 | **3.21E-02** | -4.55E-05 | [illegible] |
| chr19:44908684:T:C | rs429358 | t-tau | C | 0.228 | *APOE* | -- | Reported | 0.301 | **5.40E-161** | NA | NA | 4.77E-05 | [illegible] |
| chr2:127135234:C:T | rs6733839 | p-tau181 | T | 0.394 | *BIN1* | *BIN1* | Reported | 0.073 | **2.28E-12** | 1.184 | **6.48E-90** | 1.18E-04 | [illegible] |
| chr3:190953006:C:T | rs35327527 | p-tau181 | T | 0.369 | *GMNC* | *CCDC50* | Reported | 0.153 | **1.43E-47** | 1.009 | 3.00E-01 | 2.07E-05 | [illegible] |
| **chr7:2718675:A:G** | rs798560 | p-tau181 | G | 0.298 | *AMZ1* | *GNA12* | Novel | -0.065 | **5.81E-09** | 1.029 | **1.24E-03** | 3.73E-05 | [illegible] |
| **chr9:2197987:A:G** | rs57263785 | p-tau181 | G | 0.248 | *SMARCA2* | -- | Novel | 0.091 | **2.06E-14** | 1.014 | 1.54E-01 | 6.20E-06 | [illegible] |
| **chr11:60254475:G:A** | rs1582763 | p-tau181 | A | 0.365 | *MS4A4E* | *MS4A6A* | Novel | -0.059 | **2.94E-08** | 0.918 | **1.65E-24** | -6.30E-05 | [illegible] |
| **chr12:21279895:C:T** | rs11045930 | p-tau181 | T | 0.288 | *SLCO1A2* | *SLCO1A2* | Novel | 0.065 | **6.24E-09** | 1.01 | 2.86E-01 | 4.37E-05 | [illegible] |
| chr16:87199910:G:A | rs4843552 | p-tau181 | G | 0.409 | *C16orf95* | *MAP1LC3B* | Reported | 0.081 | **7.28E-15** | 0.982 | **3.21E-02** | -4.55E-05 | [illegible] |
| chr19:44908684:T:C | rs429358 | p-tau181 | C | 0.228 | *APOE* | | Reported | 0.318 | **3.27E-174** | NA | NA | 4.77E-05 | [illegible] |

EA: Effect Allele; MAF: Minor Allele Frequency; Numbers highlighted in bold show significance in the studies compared; significance level defined as p < 5E-[illegible] < 0.05 for AD risk and progression. Status denotes if the loci is novel or has been reported previously for the biomarker. Direction of effect has been harmon[illegible] statistics in reference to listed effect allele.

The *APOE* ε4 variant (rs429358) was the most significant variant identified across all three endophenotypes (Fig. 2A; Table 2). In addition to the *APOE* ε4 variant, rs7412, which determines *APOE* ε2 allele, and rs5117 mapped to *APOC1* within the *APOE* region were identified as independent signals for the three biomarkers.

We also replicated the previously reported *CR1* association with CSF Aβ42 (Fig. 2; Supplementary Fig. 1). The rs10779336[T] variant in the *CR1* gene region was associated with CSF Aβ42 levels and was in high linkage disequilibrium (LD) with the lead variant from Jansen *et. al.*[5] study (rs4844610[A], $r^2 = 0.95$). For CSF t-tau and p-tau181, the previously reported *GMNC* locus on chromosome 3 and the *C16orf95* locus on chromosome 16 also were also genome wide significant in this study (Supplementary Fig. 2,4,5,7)[5,11]. The lead variant rs35327527 within the *GMNC* locus from the meta-analysis, is in high LD with previously reported variants (rs35055419 $r^2 = 0.98$; rs9877502 $r^2 = 0.99$) and had a consistent direction of effect on CSF t-tau and p-tau181 (Supplementary Table 1). Similarly, the lead variant rs4843559 within the *C16orf95* locus also had high LD with the previously reported variant associated with p-tau181 in the region ($r^2 = 0.82$).

### Distinct and overlapping novel associations identified for each CSF endophenotype

In addition to the previously reported variants, we identified novel genomic loci associated with the CSF biomarkers: one locus for Aβ42, five loci for t-tau, and four loci for p-tau181 (Table 2, Fig. 2, Supplementary Fig. 1–7). Outside of a sub-threshold p-tau181 association at 8q23.1, all other p-tau181 loci were shared with t-tau.

The variant rs6733839[T] in the *BIN1* locus was significantly associated with CSF Aβ42 (Effect=-0.06; $p = 8.29\times10^{-09}$: Table 2, Supplementary Fig. 1). Although *BIN1* is a well-known AD risk locus and has been shown to affect CSF t-tau and p-tau181 levels, its direct association with CSF Aβ42 has not been reported previously[4–6,8]. This variant was also found to influence t-tau and p-tau181 levels in the meta-analysis with consistent direction of effects as in previous studies ($Effect_{t-tau}=0.06$, $p_{t-tau}=5.24\times10^{-09}$; $Effect_{ptau181}=0.073$, $p_{ptau181}=2.28\times10^{-12}$; Table 2;Supplementary Fig. 2,5; Supplementary Table 1).

The variant rs798490[A] (MAF = 0.298), located in the 7p22.3 region near *GNA12* and *AMZN1*, was associated with t-tau (Effect=-0.074, $p = 1.87\times10^{-11}$, Supplementary Fig. 2) whereas rs798560[G] (MAF = 0.298) within the same gene region, and in LD with the variant associated with t-tau ($r^2 = 0.810$), was associated with p-tau181 levels (Effect=-0.065, $p = 5.81\times10^{-09}$: Supplementary Fig. 6). An additional novel locus on 9p24.3 (rs57263785[G] near *SMARCA2*) showed significant associations with both t-tau and p-tau181 ($Effect_{t-tau}=0.087$, $p_{t-tau}=1.12\times10^{-13}$; $Effect_{p-tau181}=0.091$, $p_{p-tau181}=2.06\times10^{-14}$; Table 2, Supplementary Fig. 3, 6). The *MS4A* gene region on chromosome 11 is an important AD risk locus and has been well documented to modulate soluble triggering receptor expressed on myeloid cells 2 (sTREM2) in CSF[13]. We observed two distinct variants that were in LD ($r^2 = 0.795$) associated with t-tau and p-tau181, in this region (rs7928895[A]: $Effect_{t-tau}=-0.060$, $p_{t-tau}=5.35\times10^{-09}$, rs1582763[A]: $Effect_{p-tau181}=-0.059$, $p_{p-tau181}=2.94\times10^{-08}$: Supplementary Fig. 4, 7). Another novel association for both t-tau and p-tau181 was identified on chromosome 12 (rs11045930[T] within *SLCO1A2*;$Effect_{t-tau}=0.065$, $p_{t-tau}=3.26\times10^{-09}$: $Effect_{p-tau181}=0.065$, $p_{p-tau181}=6.24\times10^{-09}$; Supplementary Fig. 4,7). Finally, a novel genetic association on 8q23.1 near *ANGPT1* and unique to t-tau was also identified (rs1654723[A]: Effect = 0.056, $p = 4.41\times10^{-08}$, MAF = 0.411; Supplementary Fig. 3). The variant showed a suggestive association with p-tau181 ($p = 6.17\times10^{-06}$). No additional independent signals were observed in any of the novel loci.

### Cross-ancestry comparisons

We then tested whether the variants identified in participants of European ancestry were also associated in other populations.

The analysis in the non-European population revealed that, apart from the association of the *APOE* locus (rs429358) with Aβ42, no other variants reached genome-wide threshold for significance in this population (Supplementary Table 1, Supplementary Fig. 8). The association of rs429358 with t-tau and p-tau181 were also consistent with the direction observed in main analysis ($p_{t-tau}=3.17\times10^{-03}$, $p_{p-tau181}=6.25\times10^{-04}$). The variant rs10779336 in the *CR1* locus on chromosome 1 showed a nominal association with Aβ42 ($p = 1.24\times10^{-02}$). Similarly, rs6733839 in the *BIN1* locus was close to the nominal significance threshold ($p_{t-tau}= 5.53\times10^{-02}$; $p_{p-tau181}= 7.42\times10^{-02}$; Supplementary Table 1). The majority of the remaining genome wide significant variants identified in European ancestry had a consistent direction of effect size but did not even show any association in non-Europeans.

### Gene prioritization for significant loci

Using a previously validated 50-point weighted presence-absence scoring matrix[4], we performed a functional gene nomination for our significant loci. This approach utilizes evidence from variant annotation, colocalization, quantitative trait loci (QTL) overlap, and gene-based analysis as described in the methods section (Supplementary Table 2). All genes within 1 Mb up and downstream of the lead variant were evaluated (Supplementary Table 3).

We were able to nominate a functional gene for nine significant loci (Fig. 3). *CR1* was nominated as the candidate gene at the chromosome 1 locus associated with CSF Aβ42 based on evidence from colocalization as well as annotation and gene-based analysis (Fig. 3A, Supplementary Table 4,5). The locus colocalized with expression QTLs (eQTL) for *CR1* in the caudate nucleus (PP.H4 = 0.986), cerebral cortex (PP.H4 = 0.982), putamen (PP.H4 = 0.977), nucleus accumbens (PP.H4 = 0.953) and frontal cortex (PP.H4 = 0.894; Supplementary Table 4). Another gene within the complement cascade, *CR2*, also passed our gene nomination threshold (≥ 4) for prioritization but was not nominated as functional because it scored lower overall than *CR1* (Supplementary Table 3). The chromosome 2 locus, associated with all three CSF biomarkers, is the well-known *BIN1* AD risk locus, and consistently *BIN1* was nominated by the prioritization approach with no other gene in the locus scoring the required minimum (Supplementary Table 3). *CCDC50*, which is in the same 3q28

chromosomal region as *OSTN* and *GMNC*, was nominated as the functional gene for the lead variant in chromosome 3 based on the evidence of colocalization between the t-tau and p-tau181 associations and CSF protein QTL (pQTL) for CCDC50 (Fig. 3A, Supplementary Table 4). Within chromosome 7, we had two signals (one for t-tau and the other for p-tau181; rs798490[A] and rs798560[G], respectively, $r^2 = 0.810$). *GNA12* was prioritized as the candidate gene for both based on the presence of a protein altering variant (rs798488 [p.Met1Val]; $r^2 = 0.957$, Supplementary Table 5,6) and the gene-based association test (MAGMA $p = 7.46\times10^{-10}$; Fig. 3A). In this same region *AMZ1* scored second in the prioritization algorithm. The t-tau and p-tau181 signal colocalized (PP.H4 > 0.8) with *AMZ1* eQTLs in anterior cingulate cortex, cerebellar hemisphere, cortex, hypothalamus, cerebellum and nucleus accumbens (Supplementary Table 4).

The lead variant on chromosome 11 was shown to be in the *MS4A* gene region (Supplementary Fig. 3,6). We identified two different lead variants for t-tau and p-tau181, but they were in LD (rs7928895[A], rs1582763[A], $r^2 = 0.795$, respectively). Rs1582763 was previously reported to be associated with sTREM2 and mapped to the *MS4A4A* gene[13]. However, we nominated *MS4A6A* as the candidate gene for both these variants (Fig. 3A, Supplementary Table 8), which was driven by the presence of protein altering variants (rs7232 [p.Thr185Ser]; Supplementary Table 6,7) and having passed the significance threshold in the gene-based analysis (MAGMA $p = 1.53\times10^{-07}$).

We nominated *SLCO1A2* as the candidate gene for the chromosome 12 locus based on colocalization with eQTLs in the cerebellum based on GTEx v8 Brain eQTL and MetaBrain eQTL (Fig. 3A, Supplementary Table 4). *MAP1LC3B* was nominated as the candidate gene at the chromosome 16 locus because it colocalized with a CSF pQTL (Fig. 3A, Supplementary Table 4). In addition, it also had a significant QTL in both GTEx Brain eQTL and MetaBrain eQTL (Fig. 3A). *C16orf95*, which was previously reported to be a functional gene in this locus, was the second highest scoring gene but failed to pass the nomination threshold of 4 (Supplementary Table 3)[5]. It scored a total of 3.5 based on significance in gene-based analysis (MAGMA p = 5.5247e-16) and presence of significant GTEx brain eQTL associated with the gene (Supplementary Table 3). We observed that the *MAP1LC3B* locus colocalized with trans-pQTL signals for 80 unique genes including *NECTIN*, *NLGN1*, *CNTFR*, *ATP1B2* (Fig. 3B, Supplementary Table 8).

We were unable to prioritize candidate genes for the loci on chromosomes 8 and 9 because none of the tested genes passed the scoring threshold. The closest genes to the lead variants in these loci were *ANGPT1* and *SMARCA2* based on FUMA analysis. *ANGPT1* was in fact the highest scoring gene on the chromosome 8 locus even though it scored < 4 in the weighted scoring approach (Fig. 3A).

### Significant genetic loci show association with AD risk and progression

We aimed to determine whether the lead genetic variants associated with CSF AD endophenotypes have also been implicated in other AD-related phenotypes. We queried publicly available resources to identify associations with AD risk, brain amyloidosis, and AD progression[4,14,15]. Variants near *CR1* associated with Aβ42 were also significantly associated with increased AD risk (OR = 1.127, $p = 4.69\times10^{-31}$; Table 2) and brain amyloidosis (Effect = 0.097, $p = 4.75\times10^{-31}$; Table 2). Similarly, the lead GWAS variant on chromosome 2 near *BIN1* was also associated with increased AD risk (OR = 1.184, $p = 6.48\times10^{-90}$; Table 2) as well as accelerated AD progression (Effect = $1.18\times10^{-04}$; $p = 3.89\times10^{-03}$, Table 2). Lead variants in the *GNA12* locus associated with t-tau and p-tau181 were also significant for increased AD risk (OR = 1.020, $p = 2.51\times10^{-02}$ for t-tau variant; OR = 1.029; $p = 1.24\times10^{-03}$;Table 2). However, they did not appear to effect disease progression or brain amyloidosis significantly (Table 2). Two distinct variants were associated with t-tau and p-tau181 on chromosome 11 within the *MS4A* gene region (LD $r^2 = 0.795$). A recent study reported variants within *MS4A4A* (rs10897026) and *MS4A6A* (rs72918674) that modulate sTREM2 levels[16]. The lead variants from our meta-analysis were in high LD with the reported functional variants in *MS4A6A* ($r^2_{t-tau\ vs\ MS4A6A}=0.931$; $r^2_{p-tau181\ vs\ MS4A6A}=0.755$) but not with the *MS4A4A* variant ($r^2_{t-tau\ vs\ MS4A4A}=0.068$; $r^2_{p-tau181\ vs\ MS4A4A}=0.145$). In line with their associations with lower CSF t-tau and p-tau181 levels, both alleles from the analysis were also significantly associated with reduced risk of AD (t-tau variant OR = 0.928, $p = 2.08\times10^{-19}$ ; p-tau181 variant OR = 0.918, $p = 1.65\times10^{-24}$; Table 2), with 96% and 99% probability of being the causal variants within the locus for AD risk as well, based on colocalization evidence (Supplementary Table 9). However, only the allele associated with reduced t-tau levels was found to be significantly associated with slowing disease progression (Effect=$-8.97\times10^{-05}$; $p = 3.45\times10^{-02}$; Table 2). Our chromosome 16 lead variant in the *MAP1LC3B* region that was found to be significantly associated with both t-tau and p-tau181 in the meta-analysis was also significantly associated with p-tau181 in previously published CSF GWAS study (Z = 6.61, $p = 3.85\times10^{-11}$; Supplementary Table 1)[5]. However, the allele associated with increased CSF t-tau and p-tau181 allele appears to decrease the risk of AD (OR = 0.982, $p = 3.21\times10^{-02}$). Chromosome 9 loci, a known schizophrenia risk loci near *SMARCA2*, did not show significant association with AD risk or progression but was associated with brain amyloidosis (Effect = 0.033; $p = 2.25\times10^{-02}$; Table 2)[17]. Lead variants on *SLCO1A2* and chromosomes 8 loci did not show significant associations with any other phenotypes evaluated. In sum, many of the lead variants showed converging evidence across CSF biomarkers, AD risk and progression.

## Shared genetic architecture with other traits of interest

The interplay among similar genetic factors often contributes to multiple phenotypic effects[18]. Thus, by examining the relationships between complex polygenic traits we can better understand their genetic underpinnings and identify shared biological pathways. We evaluated the genetic covariance of the three CSF endophenotypes with AD risk and 19 other human-health related traits (Fig. 4, Supplementary Table 10).

We found significantly negative genetic covariance of CSF Aβ42 with AD risk ($r_{g=}$-0.013, $p_{FDR}$=$2.67\times10^{-38}$) and brain amyloidosis ($r_{g=}$-0.349, $p_{FDR}$=$2.40\times10^{-48}$). Similarly, we observed significant positive genetic covariance of CSF t-tau and p-tau181 with AD risk ($r_{g-t-tau=}$0.01, $p_{FDR}$ =$5.51\times10^{-23}$; $r_{g-p-tau181=}$0.01, $p_{FDR}$=$7.67\times10^{-25}$) and brain amyloidosis ($r_{g-t-tau}$=0.293, $p_{FDR}$=$2.62\times10^{-36}$; $r_{g-ptau181}$=0.326, $p_{FDR}$=$1.09\times10^{-45}$). These findings are consistent with established directions of effect on AD risk for these biomarkers[9].

We also observed genetic covariance between these CSF endophenotypes and other brain disorders, with all three of the endophenotypes showing significant covariance with Lewy Body Dementia (LBD; $r_{g-A\beta42}$=-0.192, $p_{FDR}$=7.34×10$^{-05}$; $r_{g-t-tau=}$ 0.130, $p_{FDR}$=1.15×10$^{-02}$; $r_{g-p-tau181}$=0.114, $p_{FDR}$=3.47×10$^{-02}$). Schizophrenia showed significant covariance with Aβ42 ($r_g$=0.025, $p_{FDR}$=6.69×10$^{-03}$). Similarly, depression and multiple sclerosis were found to have significant covariance with t-tau ($r_{g-depression}$= -0.01, $p_{FDR-depression}$=1.16×10$^{-02}$; $r_{g-sclerosis-}$=0.014, $p_{FDR-sclerosis}$=4.62×10$^{-02}$) but not Aβ42 or p-tau181. Circulating HDL levels are reported to be associated with decreased AD risk, and we observed nominally significant and positive covariance with CSF Aβ42 ($r_{g-}$=0.007, p = 1.96×10$^{-02}$) in our analysis, although this failed to pass the FDR-corrected significance threshold. Hippocampal volume was nominally associated with Aβ42 ($r_{g-}$=0.023, p = 2.58×10$^{-02}$; $p_{FDR}$=0.077) but not with t-tau and p-tau181. Exclusion of the *APOE* region had no effect on the pair-wise genetic covariance (Supplementary Table 10). In summary, our findings indicate that common genetic factors may influence both the CSF biomarkers and other neurological disorders, with AD-related traits showing stronger associations.

### PheWAS analyses

We then queried the GWAS catalog for our lead variants (excluding chromosome 19) to identify other associated phenotypes; and identified 63 trait associations that were reported for six of them (Fig. 3D, Supplementary Table 11). The majority of the associations were for the variant in the *BIN1* locus (N = 26). These included AD, dementia, cognition or similar traits (Supplementary Table 11). For the variant in the *MS4A* gene region, we found associations with 10 traits that all pointed to involvement in sTREM2 levels and AD. Lead variants on chromosomes 8, 9 and 16 were predominantly reported to be related to brain matter, with each related to a different brain region (N = 10, 5, and 3 respectively; Supplementary Table 11). Variants on chromosome 8 seem to be associated with cortical region thickness and surface area whereas variants on chromosome 9 were associated with subcortical volume. Similarly, the *MAP1LC3B* variant was found to be associated with white matter volume. At the time of the search, neither the *CR1* variant associated with Aβ42 nor the *SLCO1A2* variant associated with t-tau and p-tau181 had any reported trait associations. However, this may be a result of limitations in current catalog coverage.

#### Pathway analysis implicate lipid metabolism dysregulation independent of APOE

To better understand the biological mechanisms behind the combined effects of significant GWAS loci on the endophenotypes, we performed pathway analysis using the genes linked to independent variants in suggestively significant loci (< 10$^{-05}$) by FUMA. Genes in the *APOE* locus were not included as they tend to bias results towards lipid metabolism. Genes associated with Aβ42 were involved in immune system pathways (regulation of complement cascade, p = 8.53×10$^{-03}$), post-translational protein modification (Gamma carboxylation, hypusinylation, hydroxylation, and arylsulfatase activation, p = 1.04×10$^{-02}$; O-linked glycosylation, p = 1.54×10$^{-02}$) and infection pathways (Uptake and actions of bacterial toxins, p = 3.78×10$^{-03}$; Early SARS-CoV-2 Infection Events, p = 5.64×10$^{-03}$; Bacterial Infection Pathways, p = 1.98×10$^{-02}$; Fig. 3E, Supplementary Table 12). *CR1* and *CLU* genes were the key driver of immune-related pathways in our analysis. *FN3KRP, FN3K, GALNT8, B3GNTL1, PPP6R3* and *DYNLL1* were some of the major genes involved in post translational modification pathways. Of these genes, *DYNLL1* has been previously reported to be dysregulated in AD brain[19]. Proper post-translational modification is necessary for protein homeostasis and dysregulation of these processes may result in the accumulation of amyloid plaques and t-tau tangles.

We observed that genes associated with t-tau levels showed enrichment for lipid metabolism pathways even though genes within the *APOE* locus were excluded from this analysis (Sphingolipid metabolism, p = 3.1×10$^{-04}$; Recycling of bile acids and salts, p = 1.16×10$^{-04}$; Sphingolipid catabolism, p = 1.53×10$^{-03}$; Bile acid and bile salt metabolism, p = 1.82×10$^{-03}$, Fig. 3E, Supplementary Table 12). *SLCO1A2, SLCO1B3, SLCO1B1, B4GALT6, SMPD4, SGPP2, PPAP2A* and *ARSG* were involved in these pathways. Other pathways that showed enrichment in genes associated with t-tau included those linked to cellular transport (Transport of organic anions, p = 2.41×10$^{-05}$; Transport of vitamins, nucleosides, and related molecules, p = 1.49×10$^{-03}$), disease (Diseases of the neuronal system, p = 2.89×10$^{-03}$; Retinoid cycle disease events, p = 2.51×10$^{-03}$; Diseases associated with visual transduction, p = 2.51×10$^{-03}$) and drug kinetics (Drug ADME, p = 1.49 ×10$^{-03}$;Fig. 3E).

Pathways enriched in p-tau181-associated genes were highly correlated with those observed in t-tau with varying degrees of significance (Fig. 3E). The most enriched disease ontology terms highlighted associations with liver function markers (Bilirubin level result, $p_{tau}$=1.50×10$^{-05}$, $p_{ptau181}$= 6.32×10$^{-06}$; Bilirubin measurement, $p_{tau}$=6.61×10$^{-06}$, $p_{ptau181}$=6.02×10$^{-05,}$ Supplementary Table 12). Familial AD was also one of the top enriched disease terms in the analysis ($p_{tau}$=3.20×10$^{-05}$, $p_{ptau181}$=5.83×10$^{-05}$). Disease ontology terms enriched for genes associated with Aβ42 highlighted lifestyle factors (Physical Activity Measurement, p = 3.02×10$^{-05}$; Body Fat Distribution, P = 5.11×10$^{-05}$; Fig. 3E). REM Sleep Behavior Disorder, a significant lifestyle factor associated with neurodegeneration[20], was also significantly enriched ($p_{tau}$=6.48×10$^{-05}$; $p_{ptau181}$=7.05×10$^{-04}$; Fig. 3E, Supplementary Table 12).

Our *MAP1LC3B* locus colocalized with trans-pQTL signals for 80 unique genes in multiple chromosomes (Supplementary Table 8). Pathway analysis of all genes that had a QTL colocalize with this locus showed enrichment of neuronal development pathways (synapse assembly, p = 9.45×10$^{-09}$; axon development, p = 3.60×10$^{07}$; neuron projection morphogenesis, p = 5.49×10$^{-07}$; cell morphogenesis involved in neuron differentiation, p = 6.89×10$^{-07}$; Supplementary Fig. 9, Supplementary Table 13). This suggests that chromosome 16 locus may be contributing to the disease process through changes in neural development. Overall, we identified critical roles of immune system regulation, protein modification, lipid metabolism, and neural development pathways in AD pathophysiology.

### Discussion

Previous GWAS for CSF Aβ42, t-tau, and p-tau181 as AD endophenotypes have identified loci associated with the disease and highlighted potential disease-relevant mechanisms[5,11,12,21]. In this study, we performed a large meta-analysis using data from a total of 18,948 subjects, making this study the largest to date for these endophenotypes. We identified 12 genome-wide significant variants across all three biomarkers, eight of which were novel in their respective association. These variants were not only associated with CSF level of the three biomarkers but also showed significant association with AD risk, disease progression and brain amyloidosis (Table 2). Some of these associations were only nominally significant, however, it is important to note that we had predefined hypothesis on the expected effect of this variants. We also observed significant genetic covariance with AD risk, brain amyloidosis and Lewy body dementia for the three biomarkers.

Consistent with what has been reported previously, *APOE* locus was the most significant association in all three biomarkers[5,11,12]. Association of *APOE* with Aβ42 was the only association identified in samples from both European and non-European ancestry. Three independent signals in the *APOE* gene: 4 and 2 and an additional variant, rs5117 mapped to *APOC1* gene, were found to be associated with the three CSF biomarkers The rs5117 variant was reported to be associated with brain amyloidosis previously[22]. In addition, we also confirmed previously reported associations near *CR1* with Aβ42 along with identifying novel association near *BIN1* with Aβ42. *BIN1* is one of the most important risk locus for AD[4,8] and we observed strong colocalization of our *BIN1* locus with previously reported *BIN1* locus associated with AD risk (PP.H4 = 1; Supplementary Table 12)[4]. Further, the variant associated with CSF Aβ42 was also significantly associated with disease progression and brain amyloidosis (Table 2). Similarly, *CR1* locus colocalized with pQTL and eQTL signals from CSF and brain respectively. This evidence shows that *CR1* contributes to the disease process through both its transcriptional and translational regulation. In addition to the *CR1* gene, we propose *CR2* as an additional functional gene in this region as it was the second-highest scoring gene and passed our nomination threshold. We have previously reported an association between CR2 protein levels and AD risk[23]. In addition, *CR2*, like *CR1*, is a member of the complement system and has a role in inflammation and immune responses, both of which are associated with AD pathology[24]. Because of the similarities between the two genes, they may be acting together to influence CSF Aβ42, and by extension, AD. Not surprisingly, Pathway analysis of loci associated Aβ42 levels highlighted dysregulation of immune system pathways mostly driven by the *CR1* and *CLU* genes.

Previously reported associations on chromosomes 3 and 16 with both t-tau and p-tau181 were also confirmed[5,11]. However, we propose new functional genes *CCDC50* and *MAP1LC3B* at these loci respectively. *CCDC50* has been reported to be highly expressed in the brain and plays a role in protein clearance[25,26]. Ye *et. al.*(2024) demonstrated aggregation of endogenous *CCDC50* in human cell lines with mutant MAPT/t-tau species[25]. Their study also demonstrated that *CCDC50* readily binds to and isolates these "aggregation-prone" t-tau species promoting their degredation[25]. These observations provide support that *CCDC50* plays an integral role in the autophagic degradation of t-tau protein aggregation, which is the hallmark of AD and other neurodegenerative diseases. Similarly, *MAP1LC3B* is a major marker of autophagy[27]. Research has shown that astrocytic knockdown of *MAP1LC3B* in APP/PS1 mouse models worsened cognitive impairments while increasing t-tau phosphorylation at serine 202 and threonine 205[28]. Both loci have previously been reported to be associated with ventricular volume[5,29,30]. In addition to *CCDC50*'s role in the tau pathology, Ye *et. al.* (2024) also demonstrated that *ccdc50* knockout in mouse models led to development of hydrocephalus[25]. Further, PheWAS analysis of the lead variant in *MAP1LC3B* locus identified its associations with brain volume and white matter microstructure (Fig. 3D). In the same context, *GNA12* and *SLCO1A2* genes, which were nominated as functional for the novel chromosome 7 and 12 association respectively with both t-tau and p-tau181, have also recently been linked to be Normal pressure hydrocephalus (NPH), a condition marked by abnormal CSF buildup in brain ventricles[31]. *GNA12* has been reported to decelerate hyperphosphorylation of t-tau where as *SLCO1A2* is a known risk gene associated with progressive supranuclear palsy[32,33]. Although we were unable to nominate functional gene for chromosome 8 and 9 loci, associated with t-tau and both t-tau and p-tau181 respectively, PheWAS analysis of the lead variants in these regions revealed associations with brain morphology, cortical volume and surface area, and brain water diffusion. GNOVA analysis investigating shared genetic architecture of CSF t-tau and ptau181 levels with hippocampal volume and intracranial volume was not significant. However, this finding likely reflects lack of polygenic signal across the traits without capturing the influence of the individual variants identified in our study. Overall, our findings suggest structural alteration in brain volume may be a key mechanism associated with tau mediated AD pathology.

Besides their association with intracranial volume, the *CCDC50* and *MAP1LC3B* loci shared some additional shared characteristics. The *CCDC50* locus is reported to be pleiotropic in nature[23] and our findings suggest that the *MAP1LC3B* locus shows similar pleiotropic characteristics. It was shown to affect regulation of 80 distinct proteins across all 23 chromosomes (Fig. 3B). Majority of the genes that encoded these regulated proteins were neuronal and involved in pathways associated with synapse assembly (*FLRT3, NLGN1, SEMA4A, ADGRL3, NECTIN1, LRRC4, LRFN4*) and neuron projection (*EFNA2, EFNB2, UNC5B, NLGN1, SEMA6B, SYT1, OLFM1, FSTL4*). Further, our protein-protein interaction analysis showed that the protein product of the *CCDC50* gene was found to interact with the protein product of *MAP1LC3B* (Fig. 3C). In light of these findings, we hypothesize that these two genes play a distinct but complementary role in the regulation of t-tau and p-tau181 levels. In addition to the alteration in brain volume, disruption of the blood-brain barrier (BBB) could be another potential mechanism contributing to tau mediated disease process. *ANGPT1*, the closest gene to the chromosome 8 locus lead variant, is known to play a role in BBB integrity maintenance and *SMARCA2* near our chromosome 9 lead variant is involved in dendritic growth and morphogenesis[17,34,35]. *SLCO1A2*, functional gene at the chromosome 12 locus, encodes a sodium-independent membrane transporter and is highly expressed in human brain endothelial cells that are responsible for selective permeability of the BBB[36–38]. *SLCO1A2* has been previously reported to be associated with cortical Aβ deposition in AD[39,40]. However, its role in tau aggregation is not well understood. Induced pluripotent stem cell (iPSCs) models from familial AD showed significant downregulation of *SLCO1A2*[40]. Given the primary role of *SLCO1A2* is passive transport to the brain across the concentration gradient, we suspect that it contributes to disease process through decreased availability of substrates required for clearing tau tangle toxin in brain[40,41]. We observed that *SLCO1A2* signal from GWAS colocalized with eQTL signal from both GTEx and Metabrain in cerebellum. Cerebellum is usually though to be spared in early AD disease, with involvement being reported in later disease stage[42]. This suggests *SLCO1A2* mediated regulatory changes may occur in later disease stage in specific brain regions.

Our pathway analysis of significant loci associated with t-tau and p-tau181 showed disruption of bile and bile salt related metabolism, driven by *SLCO1A2, SLCO1B3, SLCO1B7*, and *SLCO1B1* genes. Disease ontology analysis also found bilirubin-related disease terms to be the most significant for the two phenotypes. Bile acids (BA) are potent regulators of lipids and there is growing evidence suggesting their pathogenic effect in AD through the brain-gut-microbiota (BGM) axis[43,44]. Others have reported their direct association with CSF t-tau and p-tau181 as well as PET-measured biomarkers in AD[45]. A higher level of CSF tau was associated with higher serum BA levels[45]. Disruption in lipid homeostasis is a well-researched disease mechanism in AD, however majority of the current evidence suggests these to be regulated by *APOE* gene[46]. Given our pathway analysis did not include the *APOE* gene region, we hypothesize that there is an APOE-independent axis of lipid dysregulation contributing to t-tau pathology, potentially implicating bile acid metabolism as a therapeutic target.

Another key association that may relate to lipid dysfunction in tauopathy is the novel association of variant in the *MS4A* gene region for t-tau and p-tau181 identified here. Although, the *MS4A* locus is a gene rich region, we nominated *MS4A6A* as functional at the locus based on the presence of a coding variant in the gene. *MS4A* is a well-replicated AD risk locus and genes in this region, including *MS4A6A*, are involved in sTREM2 level regulation[13]. TREM2, a member of immunoglobulin family, is expressed exclusively in microglia in Brain. Microglia are the defense cells of the brain responsible for clearing amyloid plaques and tau tangles in AD. TREM2 acts as sensor for lipids exposed during neuronal damage and are required for activation of microglial response to the injury[47].Thus, by modulating the level of TREM2, *MS4A6A* is directly responsible for mediating both protective and aggravating microglial response to neuronal insults by tau and its species. Consistently, the *MS4A6A* variant was associated with both risk of AD and disease progression.

While we were able to identify multiple novel loci associated with the phenotypes, some limitations of the study need to be considered. Despite being the largest study for these endophenotypes, we largely focus on the European ancestry population. Our sensitivity analysis with a limited number of non-European subjects highlighted the difference in direction and strength of the association identified, in line with previous studies suggesting that findings from European ancestry cannot be directly translated to other populations[48]. Thus, future research focused on other ancestries will be needed. In addition, we highlight several biological pathways in which genes associated with the phenotypes are involved. However, without additional functional analysis we cannot comment on the directionality of these changes or how these genes might mediate the effects on the highlighted pathways. Third, inclusion of subjects from individuals with disease might confound the findings as signals from disease processes rather than disease etiology might have been captured. This could be further exacerbated by the heterogeneity introduced by different subject recruitment criteria at different study sites. Fourth, our gene prioritization method focused on pQTL and eQTL resources. However, incorporating additional resources like methylation QTLs, splicing QTLs and histone QTLs could reveal additional signals that are not captured by pQTL or mQTL alone. Finally, future research to evaluate genetic correlations between Aβ42 and t-tau/p-tau181 and identify CSF Aβ42-independent genetic signals associated with CSF t-tau /p-tau181 will be needed. This would help us better understand the t-tau/p-tau181 dependent disease mechanism and help guide research on alternatives to anti-amyloid therapies.

In conclusion, this work highlights associations between novel loci and CSF Aβ42, t-tau and p-tau181 in addition to replicating findings from previous studies. The pathway analysis identified *APOE*-independent lipid metabolism dysregulation, immune system, and cellular process disturbances as potential underlying disease mechanisms. While future replication of the novel associations in larger studies will be needed, our study demonstrates the power of studying AD-related endophenotypes to better understand disease mechanisms underlying AD risk and disease progression.

### Materials and methods

## Study design

For this study, we associated individual-level genetic data with CSF biomarker levels in 18,948 unrelated European ancestry individuals from 30 different studies (Table 1). A total of 6,785 participants across 22 studies were combined into a single dataset. The summary statistics from this dataset were then meta-analyzed with summary statistics from eight cohorts encompassing 12,163 subjects. The studies that provided summary statistics are: European Alzheimer’s and Dementia Biobank (EADB, N = 7,516), The European Prevention of Alzheimer's Dementia (EPAD, N = 1,370), Ace Alzheimer Center Barcelona (ACE[49], N = 1,302), European Medical Information Framework for Alzheimer's Disease (EMIF-AD, N = 670), MISSION-AD (N = 462, Eisai), Janssen cohorts (Janssen, N = 457) and Alzheimer’s and Families cohort (ALFA + study[50], N = 386). Notably, the EADB data is a dataset from 13 different cohorts, with details provided in Supplementary Table 14.Within Janssen cohorts, two different sets of study populations were available, one from a BACEi clinical study and the other from a bapineuzumab clinical study[51,52]. More information on these studies is available in Additional Material 1. Data preparation and analysis approaches were comparable across all studies, related individuals were removed based on identity-by-descent (IBD) analysis (Supplementary Fig. 10) and only European ancestry samples were kept as identified by genetic PCA (Supplementary Fig. 10). Additional analysis was performed using subjects from populations other than those of European ancestry. In total, we had 416 subjects, including 209 African American, 63 Asian and 144 of mixed ancestry (Supplementary Fig. 10). Detailed demographic information on subjects included in this study are available in Table 1 and Supplementary Tables 14 and 15.

## CSF measurement and quality control

CSF Aβ42, t-tau and p-tau181 levels were measured using different platforms across different studies (Table 1). Detailed information on sample collection and measurement in each study has been described elsewhere[5,51–60]. CSF biomarker values are prone to technical limitations (e.g., ceiling values) and biological artifacts (e.g., cell lysis), thereby requiring quality control (QC) prior to analysis. To this end, we applied a consistent QC approach within each cohort and for each endophenotype separately. First, we performed log10 transformation of the raw biomarker values to approximate a normal distribution and outlier removal. Outliers were defined as any biomarker level lower than Q1-1.5*IQR and higher than Q3 + 1.5*IQR, where Q1 and Q3 are the first and the third quartile, respectively. Following this, we applied a z-score based data standardization (mean 0 and variance 1 within each cohort) that allows us to combine data from differing sources as continuous quantitative traits. This approach has been used extensively in previous GWAS studies and has been shown to be a robust alternative to meta-analyzing individual cohort summary statistics[11,22,54,61]. Consistent QC was performed across all contributing sites.

## Genotyping and quality control

Genotyping was performed using multiple arrays across different studies (Supplementary Table 16). Despite these differences, a consistent QC and imputation pipeline were applied across all. Detailed QC approaches within each study are presented in Supplementary Table 16 and have been previously described[5,22]. Briefly, prior to imputation, we removed SNPs and individuals with low call rate were (95% threshold in EADB, MISSION-AD and Janssen cohort; 98% threshold in remaining). Autosomal SNPs that were not in Hardy–Weinberg equilibrium ($p < 1\times10^{-6}$) were then removed. Following this, imputation was performed using the TOPMed Imputation Server (https://imputation.biodatacatalyst.nhlbi.nih.gov/; RCh38 Version R2 reference panel) for phasing and imputation of non-genotyped single-nucleotide polymorphisms (SNPs) and only variants with imputation quality ($R_{sq}$ or estimated R2) of 0.3 or greater were retained. For the 22 studies that were included in the joint analysis, QC was performed in each study individually and then combined for downstream analysis. Duplicated and related subjects were identified using IBD analysis implemented through PLINK (v2.0)[62]. To filter subjects for cryptic relatedness, Pihat > 0.2 was used as threshold (Supplementary Fig. 10). We then performed genetic PCA calculation with 1000 Genomes data as the reference cohort and retained only subjects genetically similar to the European population for downstream analysis (Supplementary Fig. 10). Variants with minor allele frequency (MAF) of 0.05% and above were kept for analysis. When summary statistics file, from contributing sites, were provided in GRCh37 genomic coordinates, the standalone Linux-based UCSC LiftOver program was used to lift them over to GRCh38 prior to analysis.

## Statistical analysis

## Single variant analysis

Genome-wide association analysis was performed using PLINK (v2.0)[62]. We utilized an additive linear regression model for each CSF biomarker. The default model included age, sex, 10 genetic PCs and cohort-array combination, where applicable, as covariates to account for common confounding factors in GWA studies. A similar approach was used to conduct GWAS analysis in subjects of non-European ancestry as a sensitivity analysis. Subjects from various non-European ancestries were included in these analyses without stratification by specific ancestries as their sample size is not large enough for multi-ethnic-meta-analyses at this time The genome wide significance threshold of $p < 5\times10^{-08}$ was used as the cutoff to define significance.

## Meta-analysis and conditional analysis

Following the single variant analysis, we meta-analyzed the summary statistics for each of the endophenotypes. We utilized the inverse variance weighted meta-analyses in METAL to perform meta-analysis[63] Only those variants that were present across 70% of the studies were kept for downstream analysis. A p-value threshold of $< 5\times10^{-08}$ was used to define genome-wide significance. A locus was considered novel if its association with the biomarker had not been previously reported or if it was not in LD with previously reported locus in the latest CSF GWAS, samples from which are also included in this study[5]. The manhattan, forest and regional associations plots were visualized using R packages “qqman” (v0.1.9), “karyoploteR” (v1.30.0) and “meta” (v7.0), along with the stand-alone software LocusZoom (v1.40) respectively[64–67]. To identify additional independent signals within the genome wide significant loci, we performed conditional analysis using GCTA-COJO[68]. We used the “—cojo-slct” parameter within the tool to identify independent signals. The combined subject-level data (N = 6785, European ancestry) were used as linkage disequilibrium (LD) reference with default COJO parameters ($p < 5\times10^{-08}$; LD window 10000 Kb, $r^2 < 0.9$) for the stepwise independent signals selection[23,30].

## Variant annotation and gene-based analysis

Variant annotation and gene-based analysis were performed using ANNOVAR and Multi-marker Analysis of Genomic Annotation (MAGMA, v1.10) tools integrated within FUMA (Functional Mapping and Annotation)[69–71]. For FUMA, variant positions were lifted over from GRCh38 toGRCh 37 at the time of this analysis. SNPs in LD with the independent significant SNPs are annotated by ANNOVAR in FUMA. Significance is defined as passing the genome-wide threshold ($p < 5\times10^{-08}$) and an LD threshold of $r^2 > 0.6$ was used. For gene-based analysis, MAGMA mapped input SNPs to a total of 18,270 genes; thus, we used $p < 2.6\times10^{-6}$ (0.05/18720) as the significance threshold.

## Colocalization

To determine if the lead AD CSF biomarker variants also contributed to signals from other molecular quantitative trait loci (QTL), we applied a Bayesian statistical framework implemented through the R “coloc” package[72,73]. We calculated posterior probabilities under the assumption of a single causal variant (“coloc.abf” function) i.e. the same variant is influencing both phenotypes. All variants within 1 Mb up and downstream from the lead variants and common in both GWAS and QTL summary statistics were used. Coloc requires that users provide three sets of prior probabilities; p1: prior probability that a variant is associated with trait 1, p2: prior probability that variant is associated with trait 2, p12: prior probability that a variant is associated with both traits. Default priors were used for both GWAS and QTL summary statistics ($p1 = p2 = 10^{-04}$; $p12 = 10^{-05}$) as this corresponds to $p < 5\times10^{-08}$ threshold to define a true association[72]. Colocalization was checked using the largest CSF protein QTL resource currently available as well as the MetaBrain eQTL and GTEx v08 Brain eQTL resources (downloaded from GTEx portal on 06/29/2022)[23,74]. If the two traits of interest had > 80% probability (H4 > = 0.8) of having the same causal variant, we considered that there is colocalization of the two traits at this locus.

## Gene prioritization

For each of the lead variants, we used a weighted presence-absence scoring matrix approach to nominate candidate genes, adapted from the one used by Bellenguez *et al*[4]. All genes within 1 Mb upstream and downstream from the lead variant were tested. Briefly, each protein coding gene is assigned a score based on evidence from the following criteria: (A) if the index GWAS locus colocalized with a QTL for a given gene. Colocalization with QTLs was assessed against two different molecular phenotypes (protein and mRNA) from three different sources as described above. (B) If the lead variant from meta-analysis is also a significant QTL in these molecular phenotypes (C) If any variant within 1 Mb up and downstream of the lead variant from the meta-analysis passes the significance threshold ($p < 5x10^{-8}$) in the QTL summary statistics, the associated gene is scored. (D) If a gene within the 1 Mb region is found significant based on MAGMA gene-based analysis. (E) If a gene is the nearest protein coding gene to the lead variant. (F) If a gene within the 1MB region has a protein-altering variant that passes suggestive significance threshold ($p < 10^{-05}$) and is in LD ($r^2 > 0.6$) with the lead variant. Each of these scoring criteria have a different scoring weight, details of which are presented in Supplementary Table 2. The maximum score that can be assigned to a gene is 50, and a minimum score of 4 is required for nomination of a candidate gene. If more than one gene in a region passes this nomination threshold, the gene with the highest overall score is nominated as the candidate gene. We did not repeat this scoring mechanism for the well-established lead variant (rs429358) that encoded the *APOE* ε4 variant on chromosome 19.

## Comparison with other AD-related endophenotypes

We leveraged three resources, each examining different aspects of AD pathogenesis, to investigate whether identified lead variants associated with Aβ42, t-tau, and p-tau181 in our study had been previously implicated in any other AD-related phenotypes. We obtained the largest AD and related dementia risk GWAS and the largest GWAS of brain amyloidosis utilizing *in vivo* amyloid positron emission tomographic (PET) imaging data [4,5,14,15]. We also looked at variants associated with disease progression by utilizing summary statistics from an in-house AD progression (defined by rate of cognitive decline) GWAS from 7,241 subjects[15]. We queried and compared the p-value for the lead variants across all these resources and compared the direction of effect sizes. Genome-wide significance threshold was used to define significance for the variants in all reference summary statistics except AD risk and progression where $p < 0.05$ was used to highlight significance. We then sought to investigate if the novel lead variants have been reported to be associated with other phenotypes. For this, we conducted a phenome-wide association (PheWAS) study using the “gwasrapidd (v0.99)” R package[75]. The “get_studies()” function within the package was used to get a studies metadata, associated with our lead variants of interest from the GWAS catalog[76] database. Additional metadata information of interest was identified through manual query of the database. The “get_associations()” function within the package was used to extract reported p-values.

## Genetic correlation and shared genetic architecture

We also sought to investigate whether the genetic factors associated with the endophenotypes showed a similar pattern among the measures or had any correlation with other complex traits. To answer this, we analyzed genetic covariance using GNOVA (genetic covariance analyzer)[77]. Covariance was assessed against AD risk and 19 other human health related traits. These included brain health-related traits such as brain amyloidosis, hippocampal volume and intracranial volume; metal health conditions like bipolar disorder, depression and schizophrenia; and other neurodegenerative disorders like frontotemporal dementia (FTD), amyotrophic lateral sclerosis (ALS) and multiple sclerosis, among others. Prior to analysis, summary statistics files were prepared using “munge.py” pipeline as recommended by the tool. Calculations were performed using default parameters. Covariance was evaluated both including and excluding *APOE* gene region. Significance was defined as FDR corrected $p < 0.05$.

## Biological inference from pathway analysis

We performed pathway analysis using the “ReactomePA” (v1.48) package in R using overrepresentation analysis[78]. Genes associated with all suggestive significant independent variants ($p < 1\times10^{-5}$) annotated by FUMA were used as input. Genes from the *APOE* region were excluded as their strong and well documented association with lipid pathways tends to skew the results and may overshadow other important associations. To further identify diseases associated with the genes of interest, we performed enrichment analysis with DisGeNET using the “DOSE” (v3.30) R package[79,80]. A $p < 0.05$ was used as significance threshold. Protein-Protein interactions (PPI) were interrogated using STRING-db[81] with a significant enrichment association defined by $p < 0.05$. The candidate genes from gene prioritization were interrogated to see if they were enriched for any interactions.

### Declarations

#### Acknowledgements

We thank all the participants and their families, as well as the involved institutions and their staff. We would like to thank UCL Genomics, London, UK, for performing the genotyping analyses of the samples within the Gothenburg H70 Birth Cohort Studies and Clinical AD Sweden. We thank the Valdecilla Biobank (PT17/0015/0019), integrated into the Spanish Biobank Network, for their support and collaboration in sample collection and management. Our heartfelt thanks to the participants of the Valdecilla Cohort for their generosity. We want to acknowledge support from Moondance Dementia Research Laboratory.

Data collection and sharing for this project was supported by The Dominantly Inherited Alzheimer Network (DIAN, U19AG032438) funded by the National Institute on Aging (NIA), the Alzheimer’s Association (SG-20-690363-DIAN), the German Center for Neurodegenerative Diseases (DZNE), Raul Carrea Institute for Neurological Research (FLENI), Partial support by the Research and Development Grants for Dementia from Japan Agency for Medical Research and Development (AMED), the Korea Health Technology R&D Project through the Korea Health Industry Development Institute (KHIDI), Korea Dementia Research Center (KDRC), funded by the Ministry of Health & Welfare and Ministry of Science and ICT, Republic of Korea RS-2024-00344521, and Spanish Institute of Health Carlos III (ISCIII). This manuscript has been reviewed by DIAN Study investigators for scientific content and consistency of data interpretation with previous DIAN Study publications. We acknowledge the altruism of the participants and their families and contributions of the DIAN research and support staff at each of the participating sites for their contributions to this study

This work was supported by grants from the National Institutes of Health (R01AG044546 (CC), P01AG003991(CC, JCM), RF1AG053303 (CC), RF1AG058501 (CC), U01AG058922 (CC), RF1AG074007 (YJS)), the Chan Zuckerberg Initiative (CZI), the Michael J. Fox Foundation ( CC), the Department of Defense (LI-W81XWH2010849), and the Alzheimer's Association Zenith Fellows Award (ZEN-22-848604, awarded to CC).

This work was supported by access to equipment made possible by the Hope Center for Neurological Disorders, the NeuroGenomics and Informatics Center (NGI: https://neurogenomics.wustl.edu/)and the Departments of Neurology and Psychiatry at Washington University School of Medicine.

This work has been supported by the ADSP Phenotype Harmonization Consortium (ADSP-PHC) which is funded by NIA (U24 AG074855, U01 AG068057 and R01 AG059716).

The ADSP-PHC cohorts include: Adult Changes in Thought (ACT, U01 AG006781, U19 AG066567), the Alzheimer's Disease Centers (ADC, P30 AG062429 (PI James Brewer, MD, PhD), P30 AG066468 (PI Oscar Lopez, MD), P30 AG062421 (PI Bradley Hyman, MD, PhD), P30 AG066509 (PI Thomas Grabowski, MD), P30 AG066514 (PI Mary Sano, PhD), P30 AG066530 (PI Helena Chui, MD), P30 AG066507 (PI Marilyn Albert, PhD), P30 AG066444 (PI David Holtzman, MD), P30 AG066518 (PI Jeffrey Kaye, MD), P30 AG066512 (PI Thomas Wisniewski, MD), P30 AG066462 (PI Scott Small, MD), P30 AG072979 (PI David Wolk, MD, PI Vivianna M Van Deerlin), P30 AG072972 (PI Charles DeCarli, MD), P30 AG072976 (PI Andrew Saykin, PsyD), P30 AG072975 (PI Julie Schneider, MD), P30 AG072978 (PI Ann McKee, MD), P30 AG072977 (PI Robert Vassar, PhD), P30 AG066519 (PI Frank LaFerla, PhD), P30 AG062677 (PI Ronald Petersen, MD, PhD), P30 AG079280 (PI Eric Reiman, MD), P30 AG062422 (PI Gil Rabinovici, MD), P30 AG066511 (PI Allan Levey, MD, PhD), P30 AG072946 (PI Linda Van Eldik, PhD), P30 AG062715 (PI Sanjay Asthana, MD, FRCP), P30 AG072973 (PI Russell Swerdlow, MD), P30 AG066506), P30 AG066508 (PI Stephen Strittmatter, MD, PhD), P30 AG066515 (PI Victor Henderson, MD, MS), P30 AG072947 (PI Suzanne Craft, PhD), P30 AG072931 (PI Henry Paulson, MD, PhD), P30 AG066546 (PI Sudha Seshadri, MD), P20 AG068024 (PI Erik Roberson, MD, PhD), P20 AG068053 (PI Justin Miller, PhD), P20 AG068077 (PI Gary Rosenberg, MD), P20 AG068082 (PI Angela Jefferson, PhD), P30 AG072958 (PI Heather Whitson, MD), P30 AG072959 (PI James Leverenz, MD), the Department of Veterans Affairs (Elaine Peskind), the Alzheimer's Disease Neuroimaging Initiative (ADNI), funded by the Alzheimer's Disease Neuroimaging Initiative (ADNI) (National Institutes of Health Grant U01 AG024904) and DOD ADNI (Department of Defense award number W81XWH-12-2-0012). ADNI is funded by the National Institute on Aging, the National Institute of Biomedical Imaging and Bioengineering, and through generous contributions from the following: AbbVie, Alzheimer's Association; Alzheimer's Drug Discovery Foundation; Araclon Biotech; BioClinica, Inc.; Biogen; Bristol-Myers Squibb Company; CereSpir, Inc.; Cogstate; Eisai Inc.; Elan Pharmaceuticals, Inc.; Eli Lilly and Company; EuroImmun; F. Hoffmann-La Roche Ltd and its affiliated company Genentech, Inc.; Fujirebio; GE Healthcare; IXICO Ltd.; Janssen Alzheimer Immunotherapy Research & Development, LLC.; Johnson & Johnson Pharmaceutical Research & Development LLC.; Lumosity; Lundbeck; Merck & Co., Inc.; Meso Scale Diagnostics, LLC.; NeuroRx Research; Neurotrack Technologies; Novartis Pharmaceuticals Corporation; Pfizer Inc.; Piramal Imaging; Servier; Takeda Pharmaceutical Company; and Transition Therapeutics. The Canadian Institutes of Health Research is providing funds to support ADNI clinical sites in Canada. Private sector contributions are facilitated by the Foundation for the National Institutes of Health (www.fnih.org). The grantee organization is the Northern California Institute for Research and Education, and the study is coordinated by the Alzheimer's Therapeutic Research Institute at the University of Southern California. ADNI data are disseminated by the Laboratory for Neuro Imaging at the University of Southern California, the Memory ang Aging Project at the Knight ADRC (Knight ADRC), supported by NIH grants R01AG064614, R01AG044546, RF1AG053303, RF1AG058501, U01AG058922 and R01AG064877 to Carlos Cruchaga. The recruitment and clinical characterization of research participants at Washington University was supported by NIH grants P30AG066444, P01AG03991, and P01AG026276. Data collection and sharing for this project was supported by NIH grants RF1AG054080, P30AG066462, R01AG064614 and U01AG052410. This work was supported by access to equipment made possible by the Hope Center for Neurological Disorders, the Neurogenomics and Informatics Center (NGI: https://neurogenomics.wustl.edu/) and the Departments of Neurology and Psychiatry at Washington University School of Medicine; the Minority Aging Research Study (MARS, R01 AG22018, R01 AG42210), the National Alzheimer's Coordinating Center (NACC, U24 AG072122),the National Institute on Aging Late Onset Alzheimer's Disease Family Study (NIA- LOAD, U24 AG056270), the Religious Orders Study (ROS, P30 AG10161, P30 AG72975, R01 AG15819, R01 AG42210), the RUSH Memory and Aging Project (MAP, R01 AG017917, R01 AG42210), and the National Institute on Aging Genetics of Alzheimer's Disease Data Storage Site (NIAGADS, U24AG041689) at the University of Pennsylvania, funded by NIA.

**LMS** is supported by NIH/NIA grants P30 AG072979; U19 AG024904;R01AG067505;DOD W81XWH2211081; he is recipient of in-kind support from Fujirebio and Roche for immunoassay reagents & equipment for the ADNI4 study.

**KS** and **AJS** are supported by grants from the National Institutes of Health (U01AG068057, U01AG072177, P30AG072976, and U19AG074879).

**MR** was supported by grants from the Bundesministerium für Bildung und Forschung (BMBF) grants No. 01GS08125 and 01GI0706.

The AIBL study has received partial financial support from the Alzheimer's Association (US), the Alzheimer's Drug Discovery Foundation, an Anonymous Foundation, the Science and Industry Endowment Fund, the Dementia Collaborative Research Centres, the Victorian Government's Operational Infrastructure Support program, the Australian Alzheimer's Research Foundation (now Alzheimer's Research Australia), the National Health and Medical Research Council (NHMRC), and the Yulgilbar Foundation. Numerous commercial interactions have supported data collection and analyses. This includes genetic data utilized in this study, which has also been supported by grants awarded by the NHMRC (GNT1161706 PI Simon Laws, GNT2001320 PI Simon Laws). In-kind support has also been provided by Sir Charles Gairdner Hospital, Cogstate Ltd, Hollywood Private Hospital, The University of Melbourne, and St Vincent's Hospital. Finally, we thank all AIBL investigators, not listed as authors, who contributed to the design and implementation of the resource but did not actively contribute data to, or participate in the development, analysis, interpretation or writing of this current study. A complete listing of AIBL investigators can be found at https://aibl.org.au/about/our-researchers/.

EPAD is supported by the EU/EFPIA Innovative Medicines Initiative Joint Undertaking EPAD grant agreement 115736. **REM** was supported by Alzheimer's Society project grant AS-PG-19b-010.

EADB: This work was supported by a grant (European Alzheimer DNA BioBank, EADB) from the EU Joint Programme, Neurodegenerative Disease Research (JPND). Amsterdam dementia Cohort (ADC): Research of the Alzheimer center Amsterdam is part of the neurodegeneration research program of Amsterdam Neuroscience. The Alzheimer Center Amsterdam is supported by Stichting Alzheimer Nederland and Stichting VUmc fonds. The clinical data-base structure was developed with funding from Stichting Dioraphte. Genotyping of the Dutch case-control samples was performed in the context of EADB (European Alzheimer DNA biobank) funded by the JPco-fuND FP-829-029 (ZonMW projectnumber 733051061).Part of the work described in this study was carried out in the context of the Parelsnoer Institute (PSI). PSI was part of and funded by the Dutch Federation of University Medical Centers and has received initial fund-ing from the Dutch Government (from 2007-2011). Since 2020, this work was carried out in the context of Parelsnoer clinical biobanks at Health-RI (https:// www. health- ri. nl/ initi atives/ parel snoer). Part of the genotyping included in this work was funded by the JPND EADB grant (German Federal Ministry of Education and Research (BMBF) grant: 01ED1619A). Research at the Belgian EADB site is funded in part by the Alzheimer Research Foundation (SAO-FRA), The Research Foundation Flanders (FWO), and the University of Antwerp Research Fund.

The work of Valdecilla was supported by grants from the Instituto de Salud Carlos III (Fondo de Investigación Sanitario, PI08/0139, PI12/02288, PI16/01652, and PI20/01011), the JPND (DEMTEST PI11/03028), the CIBERNED, and the Siemens Healthineers. The work of ADGEN was supported by EU Joint Programme—Neurode-generative Disease Research (301220) and the Academy of Finland (338182). The DELCODE study (Study-ID:BN012 ) was supported and conducted by the German Center for Neurodegenerative Diseases (DZNE). The data samples were provided by the DELCODE study group. Details and participating sites can be found at https://www.dzne.de/en/research/studies/clinical-studies/delcode. The German Dementia Competence Network (KND) is funded by the German Federal Min-istry of Education and Research (BMBF) grants Number: 01G10102, 01GI0420, 01GI0422, 01GI0423, 01GI0429, 01GI0431, 01GI0433, 04GI0434, 01GI0711. WF, SvdL, HHolstege, CT and PhS are recipi-ents of ABOARD, which is a public-private partnership receiving funding from ZonMW (#73305095007) and Health~Holland, Topsec-tor Life Sciences & Health (PPP-allowance; #LSHM20106). More than 30 partners participate in ABOARD (www. aboard- proje ct. nl). ABOARD also receives funding from de Hersenstichting, Edwin Bouw Fonds and Gieskes-Strijbisfonds.

**AR** is supported by the German Research Foundation (DFG) grants Nr: RA 1971/6-1, RA1971/7-1, and RA 1971/8-1. **ISK** was supported by the Swedish state under the agreement between the Swedish government and the county councils, the ALF-agreement (ALF 716681), the Swedish Research Council (no 11267, 825-2012-5041, 2013-8717, 2015-02830, 2017-00639, 2019-01096), Swedish Research Council for Health, Working Life and Welfare (no 2001-2646, 2001-2835, 2001-2849, 2003-0234, 2004-0150, 2005-0762, 2006-0020, 2008-1229, 2008-1210, 2012-1138, 2004-0145, 2006-0596, 2008-1111, 2010-0870, 2013-1202, 2013-2300, 2013-2496), Swedish Brain Power, Hjärnfonden, Sweden (FO2016-0214, FO2018-0214, FO2019- 0163), the Alzheimer's Association Zenith Award (ZEN-01-3151), the Alzheimer's Association Stephanie B. Overstreet Scholars (IIRG-00-2159), the Alzheimer's Association (IIRG-03-6168, IIRG-09-131338) and the Bank of Sweden Tercentenary Foundation. **SK** was financed by grants from the Swedish state under the agreement between the Swedish government and the county councils, the ALF-agreement (ALFGBG-1005471, ALFGBG-965923, ALFGBG-81392, ALF GBG-771071). The Alzheimer fonden (AF-842471, AF-737641, AF-929959, AF-939825 ). The Swedish Research Council (2019-02075, 2019-02075_15), Stiftelsen Psykiatriska Forskningsfonden, The Swedish Brain Foundation FO2024-0097. **JN** was supported by grants from the Swedish state under the agreement between the Swedish government and the county councils, the ALF-agreement (ALFGBG-971299, ALFGBG-1007553), the Swedish Alzheimer Foundation (AF-967865, AF-993736, AF-1011899), the Dementia Foundation (2021, 2023, 2024), and Stiftelsen Handlanden Hjalmar Svenssons forskningsfond (HJSV2021039, HJSV2022059, HJSV2023023). **HZ** is a Wallenberg Scholar and a Distinguished Professor at the Swedish Research Council supported by grants from the Swedish Research Council (#2023-00356, #2022-01018 and #2019-02397), the European Union's Horizon Europe research and innovation programme under grant agreement No 101053962, and Swedish State Support for Clinical Research (#ALFGBG-71320).

**MW** is supported by Forte 2016-07097. **KB** is supported by the Swedish Research Council (#2017-00915 and #2022-00732), the Swedish Alzheimer Foundation (#AF-930351, #AF-939721, #AF-968270, and #AF-994551), Hjärnfonden, Sweden (#ALZ2022-0006, #FO2024-0048-TK-130 and FO2024-0048-HK-24), the Swedish state under the agreement between the Swedish government and the County Councils, the ALF-agreement (#ALFGBG-965240 and #ALFGBG-1006418), the European Union Joint Program for Neurodegenerative Disorders (JPND2019-466-236), the Alzheimer's Association 2021 Zenith Award (ZEN-21-848495), the Alzheimer's Association 2022-2025 Grant (SG-23-1038904 QC), La Fondation Recherche Alzheimer (FRA), Paris, France, the Kirsten and Freddy Johansen Foundation, Copenhagen, Denmark, Familjen Rönströms Stiftelse, Stockholm, Sweden, and an anonymous filantropist and donor. **IS** was financed by grants from the Swedish state under the agreement between the Swedish government and the county councils, the ALF-agreement (ALFGBG-1006423, ALF965812, ALF 716681), the Swedish Research Council (2012-5041, 2015-02830, 2013-8717, 2017-00639, 2019-01096, 2022-00882), Swedish Research Council for Health, Working Life and Wellfare (2013-1202, 2018-00471, AGECAP 2013-2300, 2013-2496, 2018-00471), Konung Gustaf V:s och Drottning Victorias Frimurarestiftelse, Hjärnfonden (FO2014-0207, FO2016-0214, FO2018-0214, FO2019-0163, FO2020-0235, FO2024-0341), Alzheimerfonden (AF-554461, AF-647651, AF-743701, AF-844671, AF-930868, AF-940139, AF-968441, AF-980935, AF-1012063), Eivind och Elsa K:son Sylvans stiftelse, Ann-Louise och Sven-Erik Beiglers stiftelse. **FK** receives a postdoctoral fellowship from the Brein Instituut. **MH** wassupported by The Research Council of Finland, grant numbers 338182, Sigrid Juselius Foundation, Strategic Neuroscience Funding of the University of Eastern Finland. **VL** was supported by Strategic Neuroscience Funding of the University of Eastern Finland. **CG** and co-authors of the Karolinska Institutet AD cohort report grants from Swedish Research Council (VR) 2015-02926, 2018-02754, 2015-06799, Swedish Alzheimer Foundation, Region Stockholm County ALF-project and research school, Karolinska Institutet StratNeuro, Swedish Demensfonden, and Swedish brain foundation, during the conduct of the study. **HH** was supported by the translational research program "PHOENIX" at Sorbonne University. **NS** has received funding from the Hellenic Foundation for Research and Technology (OSCOFAI; 16472).

ACE: Authors acknowledge the support of the Agency for Innovation and Entrepreneurship (VLAIO) grant No PR067/21 for the HARPONE project. Also, the Spanish Ministry of Science and Innovation, Proyectos de Generación de Conocimiento grant PID2021-122473OA-I00, Instituto de Salud Carlos III (ISCIII), Acción Estratégica en Salud integrated in the Spanish National R+D+I Plan and financed by ISCIII Subdirección General de Evaluación and the Fondo Europeo de Desarrollo Regional (FEDER "Una manera de hacer Europa") grants PI19/00335 and PI22/01403. The support of CIBERNED (ISCIII) under the grant CB18/05/00010. The support from PREADAPT project, Joint Program for Neurodegenerative Diseases (JPND) grant No AC19/00097. The support of Fundación bancaria "La Caixa", Fundación ADEY, Fundación Echevarne and Grífols SA (GR@ACE project). **A.C**. received support from the ISCIII under the grant Sara Borrell (CD22/00125). **P.G.G.** is supported by CIBERNED employment plan (CNV-304-PRF-866). **MB** received funding from CIBERNED (Instituto de Salud Carlos III (ISCIII); EU/EFPIA Innovative Medicines Initiative Joint Undertaking, ADAPTED Grant No. 115975; EXIT project, EU Euronanomed3 Program JCT2017 Grant No. AC17/00100; MOPEAD, Innovative Medicine Initiative, Grant. Nº. 115985; PreDADQoL, ERA-NET (call 2015). Grant nº AC15/00082; TARTAGLIA (Red federada para accelerar la aplicación de la inteligencia artificial en el sistema sanitario español); PREADAPT project, Joint Program for Neurodegenerative Diseases (JPND) Grant No. AC19/00097; GECONEU Grant No. 2023-1-ELO1-KAZZ0-HED-000032173 co –founded by the European Union; Grants PI13/02434, PI16/01861, BA19/00020, and PI19/01301 from the Acción Estratégica en Salud, integrated in the Spanish National RCDCI Plan and financed by Instituto de Salud Carlos III (ISCIII)- Subdirección General de Evaluación and the Fondo Europeo de Desarrollo Regional (FEDER – "Una manera de Hacer Europa"); Fundació "La Caixa" and Grífols (GR@ACE project); and Proyectos de Investigación de Medicina Personalizada (ISCIII), PMP-DEGESCO, Grant Nº PMP22/00022.Marta Marquié received funding support from the from the European Union's Horizon 2020 research and innovation programme under the Marie Skłodowska-Curie grant agreement no. 796706 and the Instituto de Salud Carlos III (ISCIII) Acción Estratégica en Salud, integrated in the Spanish National RCDCI Plan and financed by ISCIII-Subdirección General de Evaluación and the Fondo Europeo de Desarrollo Regional (FEDER - Una manera de hacer Europa) grant PI19/00335.

**FA** receives funding from the JDC2022-049347-I grant, funded by the MCIU/AEI/10.13039/501100011033 and the European Union NextGenerationEU/PRTR. NV-T receives funding from the Spanish Research Agency MICIU/AEI/10.13039/501100011033 (grant RYC2022-038136-I cofunded by the European Union FSE+, and grant PID2022-143106OA-I00 cofunded by the European Union FEDER). Additionally, **NV-T** is supported by the William H. Gates Sr. Fellowship from the Alzheimer's Disease Data Initiative.

**Competing Interest**

**CC** has received research support from GSK and Eisai. **CC** is a member of the advisory board of Circular Genomics and owns stocks in these companies. **CC** part of the advisory board of ADmit. **DMH** is a co-founder and has equity in C2N Diagnostics. He is on the scientific advisory board of C2N Diagnostics, Denali, Genentech, Switch Therapeutics, Acta Pharmaceuticals, and Cajal Neurosciences. He consults for Pfizer, AbbVie, and Roche. **MWN, FT,** and **YW** are employees of Eisai. **HH** is an employee of Eisai Inc.; however, this article does not represent the opinion of Eisai. His contribution to this article reflects exclusively his academic and scientific expertise developed as part of an academic position at Sorbonne University, Paris, France. **HH** serves as Reviewing Editor and previously as Senior Associate Editor for the journal Alzheimer's & Dementia, the journal of the Alzheimer's Association. Part of this study was initiated and developed in line with the Alzheimer's Precision Medicine Initiative (APMI) framework. **HH** is inventor of 11 patents and has received no royalties. **HH** declares no competing financial interests related to the present article. **QSL** was an employee of Janssen Research & Development, LLC when the work was conducted, and owns company stocks. **SK** has served at scientific advisory boards, speaker and / or as consultant for Roche, Eli Lilly, Geras Solutions, Optoceutics, Biogen, Eisai, Merry Life, Triolab, Novo Nordisk and Bioarctic, unrelated to present study content. **HZ** has served at scientific advisory boards and/or as a consultant for Abbvie, Acumen, Alector, Alzinova, ALZpath, Amylyx, Annexon, Apellis, Artery Therapeutics, AZTherapies, Cognito Therapeutics, CogRx, Denali, Eisai, LabCorp, Merry Life, Nervgen, Novo Nordisk, Optoceutics, Passage Bio, Pinteon Therapeutics, Prothena, Quanterix, Red Abbey Labs, reMYND, Roche, Samumed, Siemens Healthineers, Triplet Therapeutics, and Wave, has given lectures sponsored by Alzecure, BioArctic, Biogen, Cellectricon, Fujirebio, Lilly, Novo Nordisk, Roche, and WebMD, and is a co-founder of Brain Biomarker Solutions in Gothenburg AB (BBS), which is a part of the GU Ventures Incubator Program (outside submitted work). **KB** has served as a consultant and at advisory boards for Abbvie, AC Immune, ALZPath, AriBio, Beckman-Coulter, BioArctic, Biogen, Eisai, Lilly, Moleac Pte. Ltd, Neurimmune, Novartis, Ono Pharma, Prothena, Quanterix, Roche Diagnostics, Sanofi and Siemens Healthineers; has served at data monitoring committees for Julius Clinical and Novartis; has given lectures, produced educational materials and participated in educational programs for AC Immune, Biogen, Celdara Medical, Eisai and Roche Diagnostics; and is a co-founder of Brain Biomarker Solutions in Gothenburg AB (BBS), which is a part of the GU Ventures Incubator Program, outside the work presented in this paper. **MB**has consulted for Grifols, Araclon Biotech, Roche, Biogen, Lilly, Merck, Novo-Nordisk; has served in the Advisory Boards from Grifols, Roche, Lilly, Araclon Biotech, Merck, Biogen, Novo-Nordisk, Bioiberica, Eisai, Servier, Schwabe Pharma; received fees from lectures from Roche, Biogen, Grifols, Nutricia, Araclon Biotech, Novo-Nordisk, Eisai, Terumo, Schwabe Pharma; and reports research funding from Life Molecular Imaging, Bioiberica, Grifols, Araclon Biotech, Lilly, Roche, Janssen, Alzehon, Cortyzime, Novo Nordisk, Schwabe Pharma. **MM** has consulted for F. Hoffmann-La Roche Ltd. and has served in the Spanish Scientific Advisory Board for biomarkers of Araclon Biotech. **RV**'s institution has clinical trial agreements (RV as PI) with Alector, AviadoBio, Biogen, BMS, Denali, EliLilly, J&J and UCB. **RV**'s institution has consultancy agreement (RV as DSMB or DMC member) with AC Immune and Novartis. AH has acted as a consultant for Quanterix. **CR** is founder and majority shareholder at Scottish Brain Sciences. **CR** received Compensation for study-related activities on a per-patient per-visit basis by AC Immune SA to his institution. **CR** receives consulting fees from Biogen, Eisai, MSD, Actinogen, Roche, Eli Lilly, Novo Nordisk. **SE** received research grants from EU Innovative Health Initiative (IHI), Interreg Vlaanderen-Nederland, EU Erasmus+ programme, Research Foundation Flanders (FWO), VLAIO, GSKE/FMRE (paid to institution), consulting fees from Icometrix, Novartis, Eisai (paid to institution), personal consulting fees from Roche and Biogen, personal honoraria from Eisai, Roche, travel support to institution from Biogen, Patent EP3452830B1 (institution), member of SMB/SAB for EU-H2020 project RECAGE (unpaid) and member of DSMB of PRImus-AD (unpaid), VP of Belgian Dementia Council (unpaid) and co-chair of European Alzheimer Disease Consortium (EADC) (unpaid).**LMS** has served as a consultant and on advisory boards for Biogen and Roche; has received honoraria for lectures and presentations from Biogen and Roche. **SES**. has served on scientific advisory boards on biomarker testing and clinical care pathways for Eisai and Novo Nordisk and has received speaking fees for presentations on biomarker testing from Eisai, Eli Lilly, and Novo Nordisk. **TJH** served as a Scientific Advisor for Vivid Genomics, serves as Deputy Editor for Alzheimer's & Dementia: TRCI, and serves as Senior Associate Editor for Alzheimer's & Dementia. **AL** is inventors of a filed patent application (WO2019175379 A1 Markers of synaptopathy in neurodegenerative disease) outside of the submitted work. **AL** has served as a consultant or on advisory boards for Almirall, Fujirebio-Europe, Roche, Biogen, Grifols, Novartis, Eisai, Lilly, and Nutricia, outside the submitted work. **SCJ** serves on advisory boards to AlzPath, Lilly and Enigma Biomedical. **NS** has been supported by NovoNordisk (local principal investigator in phase III international clinical trial; funding to Institution).

### Figures

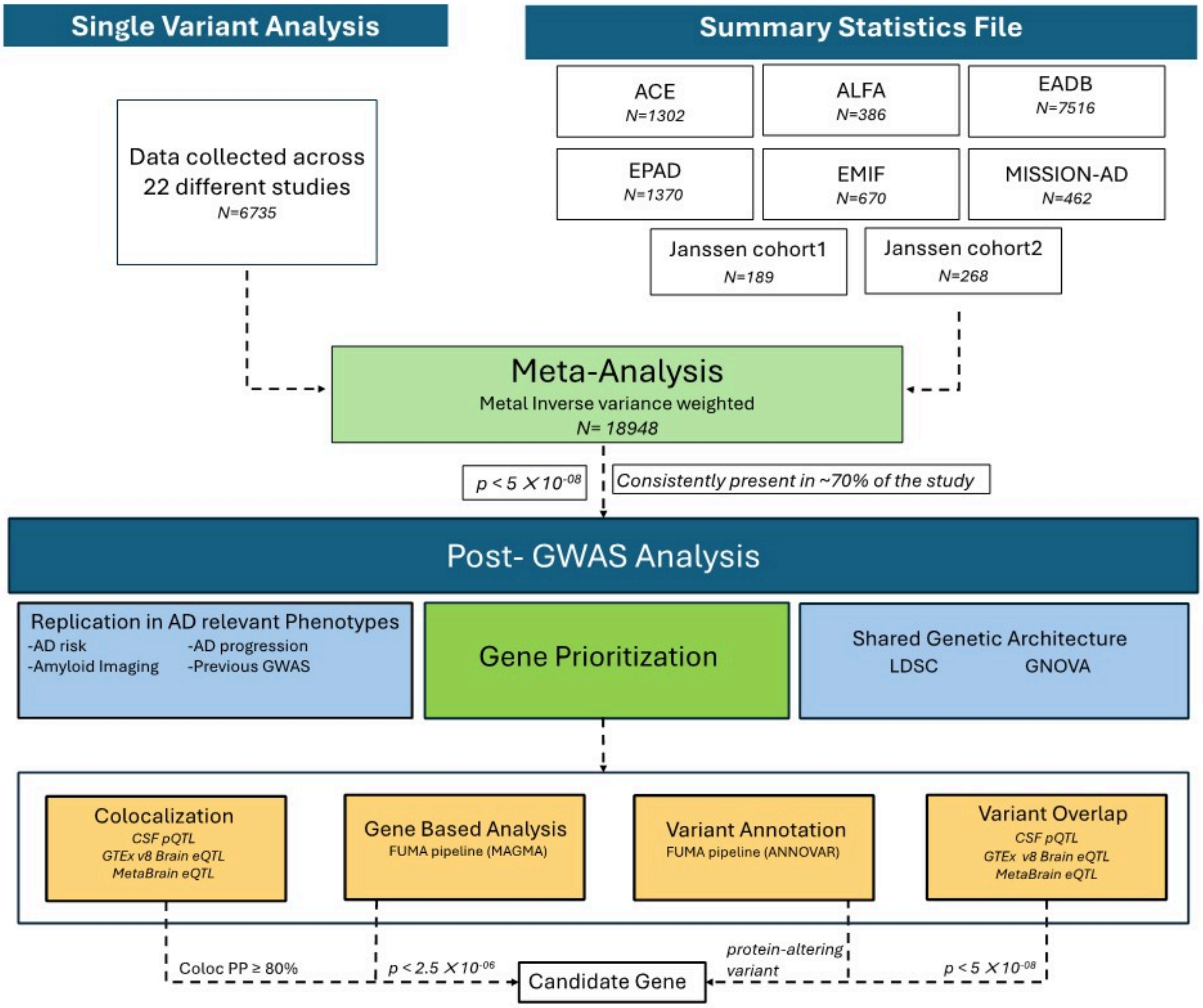

**Figure 1**

**Schematic Workflow of study design**: We meta-analyzed data across 30different studies. Raw biomarker and genetic data were collected from 22 cohorts for 6785 unique samples. Summary statistics were available from remaining sites. Consistent data preparation approach applied across all. Meta-analysis was performed using results from each individual GWAS using METAL. Significance defined $P < 5 \times 10^{-08}$. We identified replication in relevant AD traits for significant variants and checked genetic correlation with different traits. For variants that pass significance threshold, gene prioritization was done based on evidence from colocalization with quantitative trait loci (QTL) and gene/variant based functional analysis.

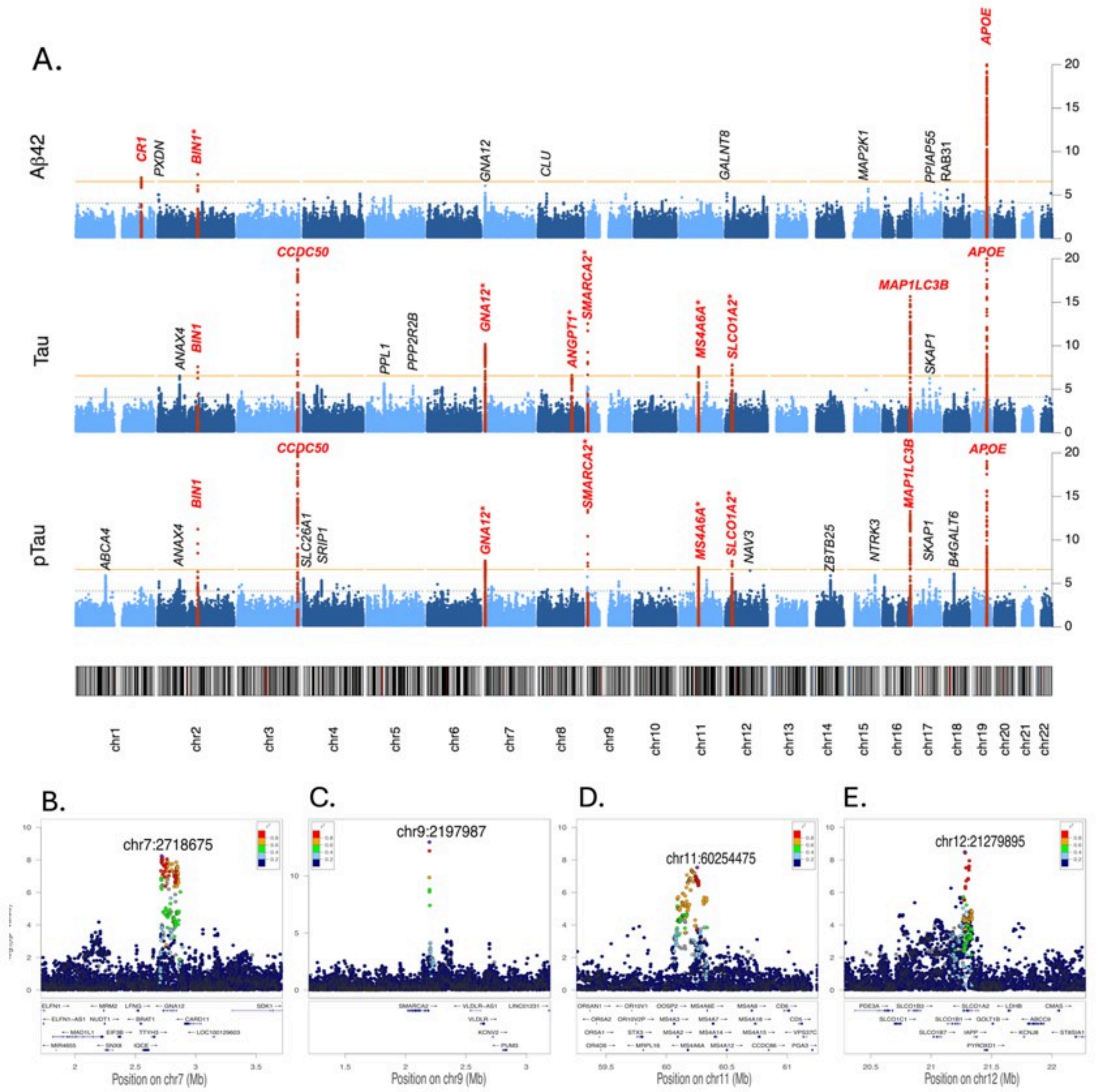

Figure 2

**Association plots from meta-analysis**. **(A)**Manhattan plot showing negative log10-transformed P values from the meta-analysis of all three CSF biomarkers. The horizontal lines represent the genome-wide significance threshold, P= 5 × 10−8 (orange) and suggestive threshold, P= 1×10−5 (grey). The loci highlighted red were those that passed genome wide threshold in each of these analyses. The genes represent genes prioritized by functional and conditional analysis for genome wide significant loci or the closet genes (shown in black) for those variants that were close to the threshold but did not pass significance. "*" denotes novel association for the trait. **(B-E)** Locuszoom plots of novel loci associated with pTau near GNA12(B), SMARCA2 (C), within MS4A gene family (D) and SLCO1A2 (E).

A.

| MarkerName | Symbol | Total | Protein Altering Variant | MAGMA | Closest Gene | pQTL-Coloc | GTEx-Coloc | MB-coloc | pQTL-Sig Locus | GTEx-sig Locus | MB-sig Locus | pQTL-Lead | GTEx-Lead | MB-Lead |
|---|---|---|---|---|---|---|---|---|---|---|---|---|---|---|
| chr1:207625371:T:G | **CR1** | 48 | 19 | 0 | 1 | 7 | 4 | 4 | 2 | 1.5 | 1.5 | 3 | 2.5 | 2.5 |
| | CR2 | 13.5 | 0 | 0 | 0 | 7 | 0 | 0 | 2 | 1.5 | 0 | 3 | 0 | 0 |
| chr2:127135234:C:T | **BIN1** | 4 | 0 | 0 | 1 | 0 | 0 | 0 | 0 | 1.5 | 1.5 | 0 | 0 | 0 |
| chr3:190953006:C:T | **CCDC50** | 7 | 0 | 0 | 0 | 7 | 0 | 0 | 0 | 0 | 0 | 0 | 0 | 0 |
| chr7:2761908:G:A | **GNA12** | 23.5 | 19 | 2 | 1 | 0 | 0 | 0 | 0 | 1.5 | 0 | 0 | 0 | 0 |
| | AMZ1 | 18 | 0 | 2 | 0 | 0 | 4 | 4 | 0 | 1.5 | 1.5 | 0 | 2.5 | 2.5 |
| chr7:2718675:A:G | **GNA12** | 20.5 | 19 | 0 | 0 | 0 | 0 | 0 | 0 | 1.5 | 0 | 0 | 0 | 0 |
| | AMZ1 | 19 | 0 | 2 | 1 | 0 | 4 | 4 | 0 | 1.5 | 1.5 | 0 | 2.5 | 2.5 |
| chr8:107381731:A:G | ANGPT1 | 2.5 | 0 | 0 | 1 | 0 | 0 | 0 | 0 | 0 | 1.5 | 0 | 0 | 0 |
| chr9:2197987:A:G | CARM1P1 | 1.5 | 0 | 0 | 0 | 0 | 0 | 0 | 0 | 1.5 | 0 | 0 | 0 | 0 |
| | KCNV2 | 1.5 | 0 | 0 | 0 | 0 | 0 | 0 | 0 | 0 | 1.5 | 0 | 0 | 0 |
| | VLDLR | 1.5 | 0 | 0 | 0 | 0 | 0 | 0 | 0 | 1.5 | 0 | 0 | 0 | 0 |
| chr11:60254475:G:A | **MS4A6A** | 21 | 19 | 2 | 0 | 0 | 0 | 0 | 0 | 0 | 0 | 0 | 0 | 0 |
| chr11:60207692:G:A | **MS4A6A** | 21 | 19 | 2 | 0 | 0 | 0 | 0 | 0 | 0 | 0 | 0 | 0 | 0 |
| chr12:21279895:C:T | **SLCO1A2** | 19 | 0 | 2 | 1 | 0 | 4 | 4 | 0 | 1.5 | 1.5 | 0 | 2.5 | 2.5 |
| | RECQL | 4 | 0 | 0 | 0 | 0 | 4 | 0 | 0 | 0 | 0 | 0 | 0 | 0 |
| chr16:87199910:G:A | **MAP1LC3B** | 10 | 0 | 0 | 0 | 7 | 0 | 0 | 0 | 1.5 | 1.5 | 0 | 0 | 0 |
| | C16orf95 | 3.5 | 0 | 2 | 0 | 0 | 0 | 0 | 0 | 0 | 1.5 | 0 | 0 | 0 |

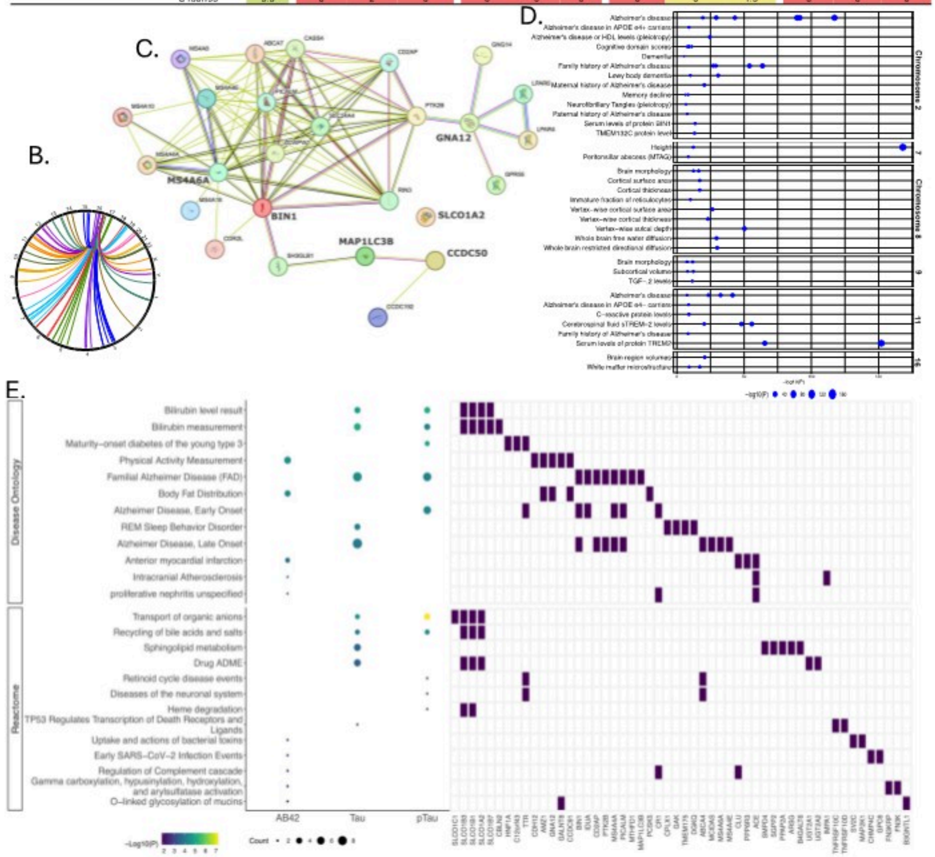

### Figure 3

Gene prioritization and downstream analysis. (A) Scoring matrix showing prioritized genes for each of our significant loci and scores from different fine mapping approaches. Genes highlighted bold passed our nomination criteria (>4 score).(B) Circos plot showing trans pQTL that colocalized with our chromosome 16 GWAS loci. (C) Protein-Protein interaction of protein products of our prioritized genes associated with t-tau and ptau.(D) Previously reported trait associated with our lead variants in GWAS loci from GWAS catalogue (E) Reactome and pathway analysis of genes associated with variants that passed suggestive significance threshold in each of our phenotype. Significance threshold was p<0.05. Y-axis represents top 5 pathways/terms associated with each phenotype. Tile plot shows genes involved in these pathways.

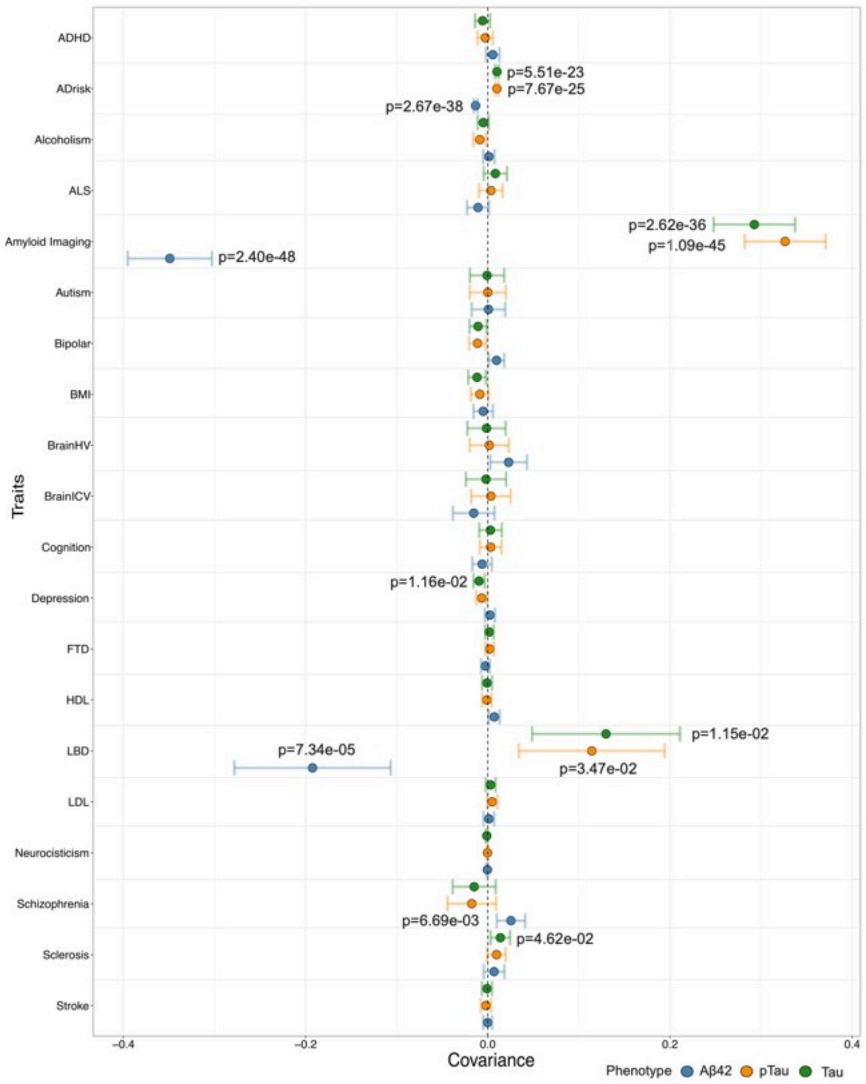

Figure 4

Dot and whisker plot showing the covariance coefficient for each phenotype across 20 different traits. Color of the dots represent each phenotype. Error bars show 95% confidence interval. FDR P-value is shown for that covariance that passed significance threshold (FDR P< 0.05).

### Supplementary Files

This is a list of supplementary files associated with this preprint. Click to download.

- F4SupplementaryFiguresCSFABTauGWAS.docx
